# The influence of bulk stoichiometry on near-ambient pressure reactivity of bare and Pt-loaded rutile TiO_2_(110)†

**DOI:** 10.1039/d4nr01702a

**Published:** 2024-08-29

**Authors:** Florian Kraushofer, Matthias Krinninger, Sebastian Kaiser, Johanna Reich, Agnieszka Jarosz, Matthias Füchsl, Gaurav Anand, Friedrich Esch, Barbara A. J. Lechner

**Affiliations:** a Functional Nanomaterials Group & Catalysis Research Center, Department of Chemistry, TUM School of Natural Sciences, Technical University of Munich Lichtenbergstr. 4 85748 Garching Germany bajlechner@tum.de; b Chair of Physical Chemistry & Catalysis Research Center, Department of Chemistry, TUM School of Natural Sciences, Technical University of Munich Lichtenbergstr. 4 85748 Garching Germany; c Institute for Advanced Study, Technical University of Munich Lichtenbergstr. 2a 85748 Garching Germany

## Abstract

The interaction of catalyst particles with reducible support materials can drastically change their reactivity. On rutile TiO_2_, processes like particle encapsulation (caused by the “strong metal–support interaction”, SMSI) have long been known to depend on the initial reduction state of the oxide. Despite this knowledge, sample stoichiometry has rarely been controlled in a reproducible manner in the surface science literature. Here, we use scanning tunnelling microscopy (STM) to explore systematically how near-ambient pressures (0.1–1.0 mbar) of O_2_, H_2_, CO and CO_2_ affect blank and Pt-loaded rutile TiO_2_(110) surfaces of different bulk stoichiometry at 600 K. To this end, we present preparation recipes that result in a sample stoichiometry always converging back to the same value, which allows us to use the same samples with constant reduction state over hundreds of preparation cycles. Comparing a highly reduced and a near-stoichiometric TiO_2_ sample, we find that surface reactivity to all four gasses differs even without Pt loading. Most surprisingly, we find that the highly reduced TiO_2_(110) is oxidized by CO_2_, but this reaction is completely inhibited on the near-stoichiometric sample. Pt nanoparticles, in turn, become encapsulated after vacuum annealing on the reduced, but not on the near-stoichiometric sample. Encapsulation on the near-stoichiometric sample is achieved only after exposing it to 0.1 mbar H_2_ at 600 K. Interestingly, we also see a further modification of the already encapsulated particles on the reduced sample under the same conditions, such that they become embedded deeper in the TiO_2_(110) surface.

## Abstract 2

The stoichiometry of rutile TiO_2_ supports determines whether platinum particles will become encapsulated, and drastically affects the TiO_2_ reactivity even in the absence of platinum.

## Graphical abstract

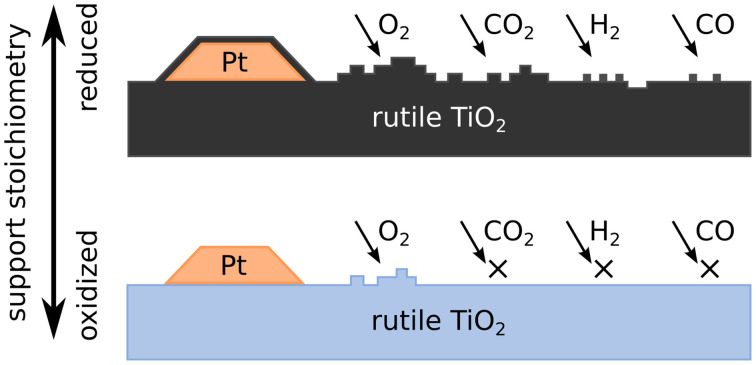

## Introduction

Rutile TiO_2_ is a prototypical support material in heterogeneous catalysis and has been a staple model system in surface science studies for many years.^1–3^ Such model studies aim to understand the atomic-scale structures and mechanisms involved in catalysis by strictly controlling sample preparation and reaction conditions. Bulk or thin film crystalline supports are first precisely characterized in ultra-high vacuum (UHV) before building up complexity to include supported metal particles and reactant molecules. However, this approach is inherently difficult to reconcile with the elevated pressures typically associated with applied heterogeneous catalysis, as many of the methods require UHV to function. This divide has been referred to as the “pressure gap”, as mechanisms and even surface structures may differ strongly between these two types of environment.^4^ In recent years, this issue has been somewhat alleviated as technical advances have allowed classical UHV-based surface-science methods, such as X-ray photoelectron spectroscopy (XPS) and scanning tunnelling microscopy (STM), to also be applied in the “near-ambient pressure” (NAP) regime, *i.e.* up to some mbar.^5–12^

In the case of TiO_2_, its interaction with supported metal particles is particularly complex under reducing conditions. A strongly reduced capacity to interact with molecules from the gas phase, and thus significantly lower activity for most reactions, was reported by Tauster for metal catalysts on reducible oxides, and termed the “strong metal–support interaction” (SMSI).^13,14^ After some initial debate whether this effect was primarily electronic (through changed particle charge) or geometric (through blocking adsorption sites) in nature, it is now the accepted explanation that the particles become encapsulated by a thin suboxide (TiO_*x*_) layer.^15–21^ One of the most convincing demonstrations was provided in an STM study by Dulub *et al.*, who obtained atomic resolution of such an overlayer on large Pt particles after UHV annealing, exhibiting a “zigzag” structure.^16^ Similar films can be obtained by depositing titanium on Pt(111) and annealing in oxygen, confirming the assignment as TiO_*x*_ rather than a Pt–Ti surface alloy.^22,23^ In analogy to Dulub *et al.*,^16^ Bowker *et al.* showed that both a “zigzag” and a “pinwheel” overlayer structure can coexist on encapsulated Pd on strongly reduced rutile TiO_2_(110).^18,19^ Recently, it was shown that a different type of encapsulation by stoichiometric TiO_2_ occurs in oxidizing environments,^21,24–26^ and that particles can even be de-encapsulated in certain H_2_ : O_2_ mixtures.^25^ Such reaction mixtures may arguably be the more relevant conditions for many catalysts, but have only recently become widely accessible to model system investigation due to the advances in the NAP-XPS and NAP-STM methods mentioned above.

While SMSI-induced encapsulation and de-encapsulation dynamics clearly depend on the gas environment and temperature, it has further been reported that the sample history, and in particular the bulk stoichiometry of TiO_2_ samples, plays a decisive role.^13,27^ While UHV-prepared TiO_2_(110) surfaces exhibit oxygen vacancies (V_O_) as the primary defect, Ti interstitials (Ti_int_) are known to be the relevant defects in bulk rutile,^28^ and can reach concentrations of up to *x* ≈ 4 × 10^−4^ in TiO_2−*x*_, which corresponds to one Ti_int_ per 1250 unit cells.^29^ Bulk stoichiometry can be roughly estimated by the sample colour, which turns from transparent yellow-white when stoichiometric *via* translucent blue when somewhat reduced to oblique black when strongly reduced.^1^ Reducing a sample further leads to the formation of linear defects of Ti_2_O_3_ stoichiometry on the (110) facet, which accumulate to form a (1 × 2) surface reconstruction at higher coverage.^30–32^ Reducing the sample even further induces the formation of crystal shear planes in the bulk.^28,29,33^

Interestingly, it has been shown that the encapsulation of particles depends on the electronic structure of the support. Rutile TiO_2_ typically acts as an n-type semiconductor due to self-doping with Ti_int_ defects. Fu *et al.* studied the encapsulation of Pd nanoparticles and found that encapsulation occurs on strongly reduced, but not on near-stoichiometric samples.^20^ However, encapsulation could also be achieved on Nb-doped samples even when they were only lightly reduced,^20^ which indicates that the relevant difference between the samples lies in their electronic structure, rather than just the availability of excess Ti.

The effect of sample stoichiometry on the interaction with clusters or nanoparticles was already considered in some previous publications.^34,35^ However, in these works, “low reduced” or “oxidized” samples were obtained either by following a reducing preparation of sputtering and annealing in ultra-high vacuum (UHV), but simply performing fewer preparation cycles than usual,^34^ or by reoxidizing only the surface of a reduced sample at low temperature.^35^ Neither approach yields a reproducible reduction state, as continued preparation cycles will keep reducing the bulk. The latter approach of low-temperature surface reoxidation has the added disadvantage of introducing a significant stoichiometry profile near the surface, which is both hard to control and to quantify.

Model catalyst studies generally aim to explore catalyst-support interactions in a variety of environments and at different metal loadings. Between experiments, sputtering and annealing cycles are required to remove the added metal particles, and to otherwise clean the samples. To control for the effect of sample stoichiometry, it is important that over tens or even hundreds of these preparation cycles, the samples always converge back to a reproducible bulk stoichiometry. It is easy to see that this is impossible to achieve for a TiO_2_ sample without oxygen annealing, as each sputtering step will reduce the near-surface region. In contrast, a typical approach used to “reoxidize” the TiO_2_ surface is through UHV annealing. This works for the surface region because Ti_int_ can be accommodated in the bulk,^28^ but the resulting bulk concentration of Ti_int_ scales approximately linearly with the number of preparation cycles, and so samples will become more bulk reduced over time. If samples are instead exposed to a mildly oxidizing annealing step after each time they were sputtered, Ti_int_ can diffuse back to the surface and react with oxygen from the gas phase to form new TiO_2_ layers.^36^ Although this is an activated process,^36–38^ it is obvious that the rate of reoxidation partly depends on the bulk Ti_int_ concentration. It follows trivially that alternating between sputtering and oxygen annealing will always lead to convergence at some fixed bulk reduction state, although if the reoxidation is too mild, the converged limit may still be a heavily reduced state.

Here, we report preparation recipes yielding rutile TiO_2_(110) crystals with reproducible stoichiometry and explore how sample stoichiometry affects their reactivity. Following previous work, we refer to the two differently prepared samples as lightly reduced and heavily reduced (LR-TiO_2_ and HR-TiO_2_, respectively).^34^ We then use NAP-STM to study the reactivity of blank and Pt-loaded LR-/HR-TiO_2_(110) surfaces to near-ambient pressures (0.1–1.0 mbar) of O_2_, H_2_, CO, and CO_2_ at 600 K. We deposit smaller amounts of platinum than in previous studies to avoid interconnected films^18^ or partially buried “iceberg” structures,^16^ and to better observe particle dynamics. The interpretation of our microscopy results is supported by low energy electron diffraction (LEED), temperature programmed desorption (TPD), (NAP) X-ray photoelectron spectroscopy [(NAP-)XPS], low energy ion scattering (LEIS) and Auger electron spectroscopy (AES). Even without a cocatalyst, we find strong surface interactions of HR-TiO_2_ with all four gasses, including significant surface oxidation by CO_2_. Platinum nanoparticles on this surface exhibit an SMSI-induced TiO_*x*_ overlayer after UHV preparation,^15,16,18^ and are remarkably dynamic in a NAP H_2_ atmosphere at 600 K. In contrast to HR-TiO_2_, the near-stoichiometric LR-TiO_2_ sample shows no interaction with CO_2_, H_2_ or CO, confirming our premise that strict control of the sample stoichiometry is required. Pt particles on LR-TiO_2_ initially remain unencapsulated under UHV annealing, but encapsulate when the surface is reduced in a NAP H_2_ atmosphere at 600 K.

## Methods

### Sample preparation and equilibration

TiO_2_(110) single crystal samples were obtained from SurfaceNet GmbH and subjected to cleaning cycles of sputtering (1 keV Ar^+^ ions) and annealing. Cleanliness was regularly checked by AES (in the UHV system used for NAP-STM) or XPS (in the UHV systems used for TPD and LEIS measurements). Two of the four samples initially showed traces of potassium impurities, which could be depleted by repeated cleaning cycles. In later experiments, no elements aside from O, Ti, Pt and Ar (implanted during sputtering, also sometimes visible in STM) were ever detected.

**Fig. 1:**
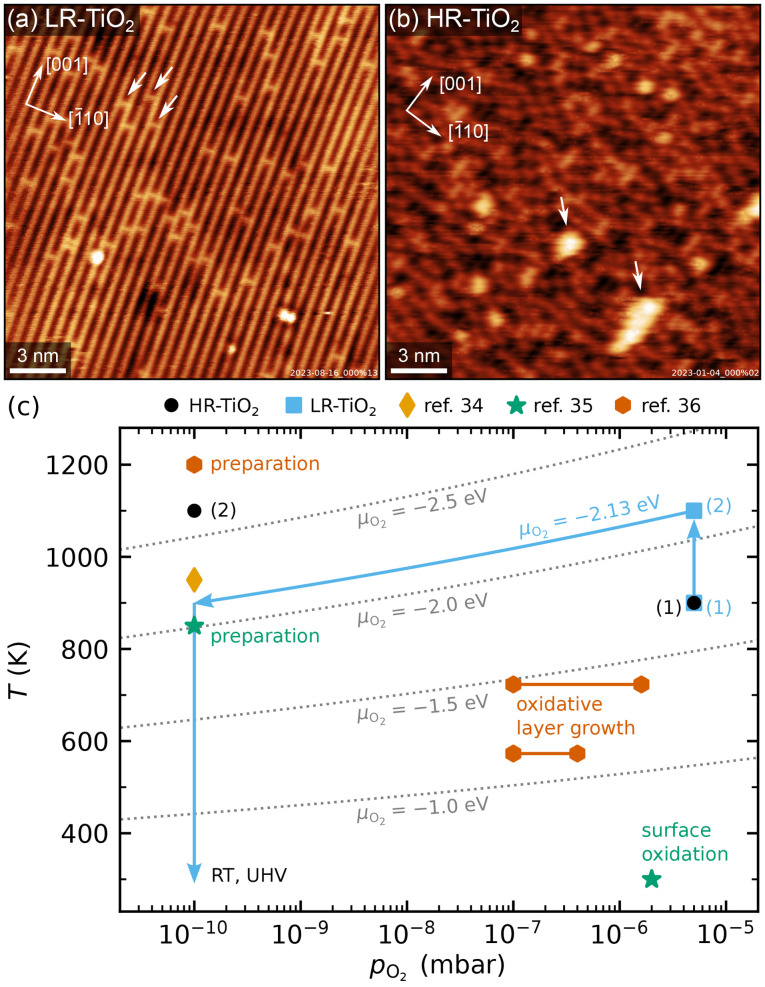
(a and b) Representative STM images of as-prepared rutile TiO_2_(110) surfaces, showing (a) the LR-TiO_2_ sample (*U*_sample_ = 1.2 V, *I*_tunnel_ = 0.2 nA) and (b) the HR-TiO_2_ sample (*U*_sample_ = 1.2 V, *I*_tunnel_ = 0.2 nA). White arrows in (a) mark oxygen vacancies, while white arrows in (b) mark (1 × 2)-like line defects. The crystal directions are given in the top left corners, respectively. (c) Pressure-temperature diagram showing the preparation conditions for LR-TiO_2_ (blue squares) and HR-TiO_2_ (black circles), as well as preparation and experimental conditions from ref. 34–36. Dotted grey lines mark constant oxygen chemical potential *μ*_O_2__.

We applied two different preparation recipes to obtain samples with distinct, well-controlled stoichiometries. Typical STM images of the as-prepared TiO_2_(110) surfaces are shown in Fig. 1, and additional larger-area images are shown in Fig. S1.† In both cases, preparation was carried out with fully automated pressure and temperature control, which let us achieve perfectly reproducible conditions. Fig. 1(c) shows the oxygen pressures and temperatures used to prepare the two samples discussed here (blue squares and black circles), as well as preparation conditions from ref. 34–36. For ref. 35 and 36, the experimental conditions for surface reoxidation are also indicated.

The more strongly reduced sample (HR-TiO_2_) was first prepared in a classical manner by cycles of sputtering and annealing in UHV at 1100 K. Note that by “heavily reduced”, we mean samples with maximal Ti_int_ concentration before the onset of structural changes of surface or bulk, rather than (1 × 2)-reconstructed surfaces. Once the sample turned completely black and first surface defects indicating the onset of the (1 × 2) reconstruction^30–32^ appeared [bright features marked by white arrows in Fig. 1(b)], we stabilized the stoichiometry by introducing an additional oxygen annealing step. For all subsequent experiments, the preparation consisted of sputtering for 15 minutes, then annealing in 3 × 10^−6^ to 5 × 10^−6^ mbar O_2_ for 10 minutes at 900 to 950 K, and finally annealing in UHV at 1100 K for 20 minutes [black circles (1) and (2) in Fig. 1(c)]. Since the reduced surface defects visible in STM give a reliable indication of at least the near-surface stoichiometry,^39^ for this sample, we occasionally varied the temperature and pressure of the oxygen annealing step to more quickly arrive back at the desired state. This was particularly helpful after the strongly oxidizing experiments, as will be discussed below. The bright defects were present in small amounts on the HR-TiO_2_ sample during all experiments described below. They always bridge two Ti rows and are the smallest moiety of the Ti_2_O_3_-stoichiometric reconstruction formed on strongly reduced samples, which becomes (1 × 2)-periodic at high coverage.^30–32^ The same type of defect also appears as a metastable phase during sample reoxidation, where it forms “crosslinked” (1 × 2) structures before being transformed into new (1 × 1) layers [orange hexagons in Fig. 1(c)].^32,36,40^

For the only lightly reduced sample (LR-TiO_2_), the stoichiometry can in turn be estimated by the number of surface oxygen vacancies V_O_ [bright spots bridging two Ti rows, marked by white arrows in Fig. 1(a)]. However, they are harder to quantify quickly and routinely, because other defects can have a similar appearance in STM, and acquiring images with sufficiently high quality can be time-consuming. Therefore, we instead consistently used the same preparation recipe and let the stoichiometry equilibrate over a large number of cycles. Each cycle consisted of sputtering for 10 minutes, then annealing in 5 × 10^−6^ mbar O_2_ for 20 minutes at 900 K, and for 10 minutes at 1100 K [blue squares (1) and (2) in Fig. 1(c)]. The low-temperature annealing step has the purpose of reoxidizing the sample, but we found that a high-temperature annealing step is also required to obtain nicely flat (110) surfaces. At 1100 K, the oxygen pressure is likely insufficient to induce significant reoxidation, but should still suppress thermal reduction.

The crystal remained light blue and translucent throughout all experiments shown here, even over a total of hundreds of preparation cycles. Sample conductivity remained high enough for STM and AES throughout, but after some initial preparation cycles (possibly depleting a low concentration of natural dopants), we observed charging effects in LEED at room temperature (RT) below an incident electron energy of 200 eV.

One additional point to consider in the preparation of LR-TiO_2_ is that when samples are cooled down in oxygen, Ti_int_ react with O_2_ to form undesired TiO_*x*_ defects. This behaviour has previously been studied as a function of oxygen pressure and temperature.^36–38^ The conditions investigated in ref. 36 are indicated by the orange hexagons labelled as “oxidative layer growth” in Fig. 1(c). These TiO_*x*_ species typically form more quickly than they can be incorporated into new (1 × 1) terraces, so this pressure and temperature region should be avoided when cooling down the samples. At even lower temperatures, oxygen reacts with V_O_, healing the vacancy and yielding an additional on-top oxygen [O_ot_, green star in Fig. 1(c)].^35^ However, pumping out the oxygen while the sample is still at 1100 K may instead introduce additional V_O_ defects through thermal reduction. Either species is undesirable if the goal is for the surface to reflect the bulk stoichiometry. We solved this issue by automatically ramping our oxygen pressure to keep the oxygen chemical potential *μ*_O_2__ constant during the cooling ramp. The final annealing step at 1100 K and 5 × 10^−6^ mbar of a pure oxygen atmosphere corresponds to *μ*_O_2__ = −2.13 eV.^41^ Keeping this value constant during a linear temperature ramp yields an approximately linear ramp of log(*p*_O_2__), reaching the base pressure of 1 × 10^−10^ mbar at 900 K, as indicated by the blue arrows in Fig. 1(c). Where automatic sample preparation is unavailable, a similar result could likely be obtained by cooling the sample to 900 K in oxygen, then pumping to UHV before continuing the cooling ramp.

### Experimental methods

Experiments were performed in three independent UHV setups, using two separate pairs of LR-TiO_2_ and HR-TiO_2_ samples.

Most data were acquired in a system consisting of two chambers with a base pressure of <1 × 10^−10^ mbar. One of the chambers houses an SPM Aarhus 150 NAP instrument (SPECS), where all NAP-STM data were acquired. STM was performed in constant-current mode, using electrochemically etched tungsten tips. The other chamber contains instruments for LEED (ErLEED 150, SPECS) and AES (DESA 150, Staib Instruments), a sputter gun (IQE 11, SPECS) and an electron-beam heater, as well as an electron-beam evaporator (EBE-1, SPECS) for depositing Pt (Goodfellow, 99.95%) and a quartz-crystal microbalance (OmniVac) to calibrate the deposition rate. Samples were mounted on stainless steel plates, and their temperature was measured by a K-type thermocouple pressed to the back of the sample by a spring. Since we also heat the samples from the backside, this may result in a slight overestimation of the actual surface temperature, although the effect should be small as the temperature is equilibrated over many minutes. All gasses used in NAP-STM and NAP-XPS experiments were acquired from Westfalen AG (H_2_, O_2_: grade 5.0, CO_2_: grade 4.5, CO: grade 3.7). H_2_ and CO were additionally cleaned using a liquid nitrogen cold trap.

LEIS and NAP-XPS measurements were performed on the same samples, which were initially prepared in the NAP-STM setup and then transferred in-house *via* a vacuum suitcase (*p* < 5 × 10^−9^ mbar) to the NAP-XPS setup (base pressure <5 × 10^−10^ mbar). Samples were heated from the back using a laser heater (OsTech DioSource, 976 nm). XPS data were acquired with a PHOIBOS 150 NAP hemispherical analyser (SPECS) and a monochromated X-ray source (XR 50 MF with μFOCUS 600, SPECS). The same analyser and a scannable ion source (IQE 12, SPECS) were used for LEIS.

TPD and corresponding STM and XPS measurements were performed in a separate UHV setup consisting of two chambers with a base pressure of <1 × 10^−10^ mbar. In one chamber, C^18^O (Eurisotop, 96.1%) TPD was measured with a quadrupole mass spectrometer (QMA 200 Prisma Plus, Pfeiffer Vacuum) contained in a “sniffer” device^42^ described in detail elsewhere.^43^ An Omicron EA125 hemispherical analyser and a SPECS XR 50 X-ray source were use for XPS. Pt (Goodfellow, 99.95%) was deposited from a FOCUS EFM-3 electron-beam evaporator. Samples were heated using a pyrolytic boron nitride heater located directly below the sample, while the temperature was measured with a type K thermocouple pressed onto the rim of the tophat-shaped samples. In the other chamber, STM was measured with a Scienta-Omicron VT-AFM instrument in constant-current mode using electrochemically etched tungsten tips, to ensure the sample stoichiometry and surface structure of the two samples is comparable in both setups.

Both the NAP-STM and TPD vacuum systems, where sample preparation was performed, are equipped with automated sputtering, temperature and pressure control systems, which allows running a large number of preparation cycles, typically during the night between experiments. This also enabled linking pressure and temperature setpoints to regulate to a specific oxygen chemical potential *μ*_O_2__, which we applied to avoid non-equilibrium oxidation or reduction during the cooling ramps.

STM images were corrected by row alignment along the slow-scan direction and subsequent plane subtraction. Mean apparent nanoparticle heights were determined by evaluating the mean height of their top facet with respect to the supporting TiO_2_ terrace, averaging over 30–80 particles in each experiment.

## Results

### Reactivity of blank TiO_2_ surfaces

**Fig. 2:**
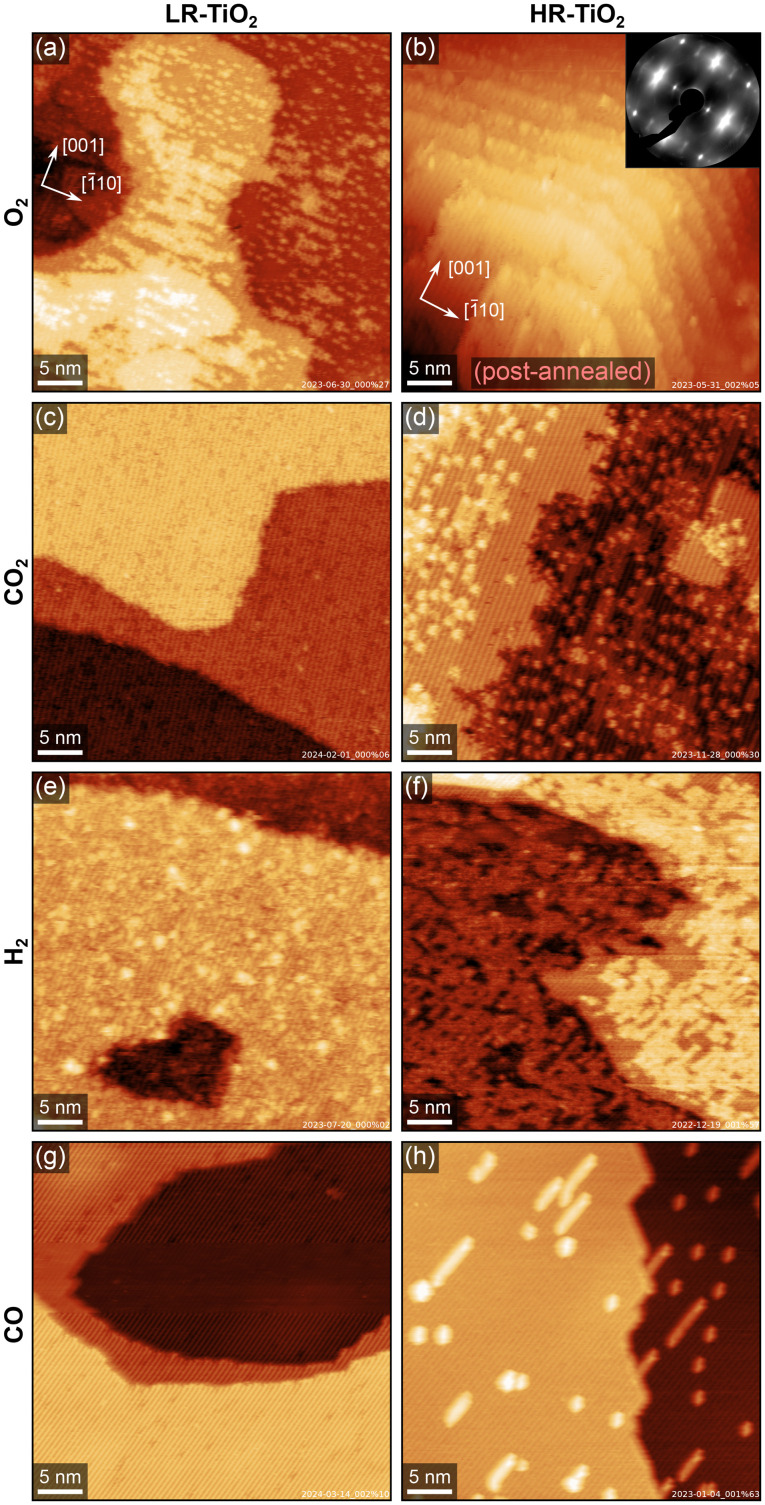
Representative STM images of LR-TiO_2_ (left) and HR-TiO_2_ (right) exposed to NAP atmospheres of various gasses at 600 K. The crystal directions of the two crystals are given in the top left corners of (a and b), respectively. (a) LR-TiO_2_ after exposure to 0.1 mbar O_2_ at 600 K for 15 minutes, image acquired at RT in UHV. (b) HR-TiO_2_ after exposure to 0.1 mbar O_2_ at 600 K for 15 minutes, image acquired at RT in UHV after post-annealing at 1100 K for 10 minutes in UHV. The inset shows a LEED image taken before post-annealing. (c) LR-TiO_2_ after exposure to 1 mbar CO_2_ at 600 K for 30 minutes, image acquired at RT in UHV. (d) HR-TiO_2_ after exposure to 1 mbar CO_2_ at 600 K for 30 minutes, image acquired at RT in UHV. (e) LR-TiO_2_ after exposure to 1 mbar H_2_ at 600 K for 90 minutes, image acquired at RT in UHV. (f) HR-TiO_2_ in 1 mbar H_2_ at 600 K, image acquired in gas atmosphere after 100 minutes. (g) LR-TiO_2_ in 0.1 mbar CO at 600 K, image acquired in gas atmosphere after 40 minutes. (h) HR-TiO_2_ in 1 mbar CO at 600 K, image acquired in gas atmosphere after 40 minutes. Scanning parameters *U*_sample_ and *I*_tunnel_ were (a) 2.0 V, 0.2 nA, (b) 1.2 V, 0.01 nA, (c) 1.7 V, 0.2 nA, (d) 1.6 V, 0.6 nA, (e) 1.3 V, 0.1 nA, (f) 1.3 V, 0.2 nA, (g) 1.4 V, 0.3 nA, (h) 1.0 V, 0.5 nA.

Fig. 2 summarises the results of exposing the LR-TiO_2_ and the HR-TiO_2_ samples to various gasses at 600 K and 0.1–1.0 mbar. We show representative stable and well-resolved STM images for each condition, selecting measurements either under NAP conditions or after the reaction, depending on *in situ* imaging stability under the respective conditions. We observed no significant changes during pumping and cooling, *i.e.* while *in situ* image quality is lower, images acquired near the end of exposure correspond well to the higher-quality images acquired in UHV.

The effect of 0.1 mbar of oxygen is similar to what was observed previously at lower pressures (≤2 × 10^−6^ mbar),^31,32,36,40^ and can be most clearly observed on the LR-TiO_2_ sample [Fig. 2(a), a higher-magnification STM image is shown in Fig. S2(a)†]. Ti_int_ from the bulk reacts with oxygen from the gas phase, forming bright point defects which are reported to be a precursor species to the line defects associated with the (1 × 2) reconstruction.^36^ When these defects accumulate, they can form patches of a “crosslinked” (1 × 2) phase,^40^ or a more amorphous, metastable “rosette” phase at lower temperature.^31,32^ It has been proposed that these crosslinks shift the stoichiometry of the (1 × 2) reconstruction from Ti_2_O_3_ closer to TiO_2_ by incorporating bridging oxygen atoms.^32,40^ Once small patches of such intermediate phases have formed, these are then transformed into new (1 × 1)-periodic terraces, presumably with bulk-like TiO_2_ stoichiometry and termination.^32,40^ The bright patches seen in Fig. 2(a) and Fig. S2(a)† appear similar to the smaller, more amorphous structures reported to form at ≈500 K in low oxygen pressure,^31,32^ rather than the larger, (1 × 2)-periodic structures reported at 670–830 K.^36,40^

Post-annealing the same sample at 800 K in UHV for 10 minutes entirely removes the isolated point defects, as shown in Fig. S2(b and c).† However, we still observe some bright linear features extended along [1̄10], *i.e.* perpendicular to the titanium rows, as well as poorly ordered patches with slightly lower apparent height than the added (1 × 1) terraces [Fig. S2(c)†], again similar in appearance to the “rosette” phase.^31,32^ We tentatively propose that the linear features are structurally related to this phase, with intermediate stoichiometry TiO_*x*_ (1.5 < *x* < 2), and are only prevented from taking a more stable shape by diffusion barriers.

On the HR-TiO_2_ sample, we were unable to obtain meaningful STM images during or even directly after exposure to 0.1 mbar O_2_ at 600 K, likely due to high surface roughness and poor ordering. The STM image shown in Fig. 2(b) was obtained after post-annealing the sample at 1100 K in UHV for 10 minutes. While the surface termination is mostly (1 × 1) after this treatment, STM still shows strongly increased surface roughness and a correspondingly high step density. Note that while the step density may vary on different areas of the same sample, step bunching as seen in Fig. 2(b) was never observed on as-prepared samples in this work. Conversely, the high step density after O_2_ treatment is not limited to this single image, but is representative of other spots on the same sample; we were unable to find any larger flat terraces. We note again that this is already after post-annealing at 1100 K, which usually results in rather flat surfaces (see *e.g.* Fig. S1†). A LEED image taken before the post-annealing step is shown in the inset to Fig. 2(b) [larger size in Fig. S3(a)†]. Many of the LEED spots exhibit highly diffuse profiles, as well as streaking along the unit cell diagonals, providing further evidence for a highly disorganized surface. A LEED image taken after the post-annealing step, corresponding to the STM image shown in Fig. 2(b), is shown in Fig. S3(b).† While the sharpness of the spots is somewhat improved, streaking along the unit cell diagonals is still pronounced. This can be understood as a signature of the periodically spaced steps also seen in STM.

Flat surfaces could be recovered through the standard preparation cycles, but it is worth noting that we often observed screw dislocations and half-steps indicative of bulk shear planes^28,29,33^ on HR-TiO_2_ following NAP O_2_ experiments. Although these defects were eventually healed over many re-preparation cycles, this suggests that at 0.1 mbar, the reoxidation proceeds too quickly for perfectly crystalline growth.

STM images of both TiO_2_ samples after exposing them to 1 mbar CO_2_ for 30 minutes at 600 K are shown in Fig. 2(c and d). On the HR-TiO_2_ sample [Fig. 2(d), higher-magnification image in Fig. S4(b)†], the resulting surface appears qualitatively similar to that obtained after O_2_ exposure on LR-TiO_2_ [Fig. 2(a)], and the ones reported in the literature for oxidation in low O_2_ pressure.^31,32,36,40^ A large number of bright point defects (likely TiO_*x*_ precursor species) coexists with linear (1 × 2)-like defects, small interlinked (1 × 2)-like patches, and small terraces of a (1 × 1)-periodic surface. An STM image acquired in the gas atmosphere only 10 minutes after reaching 600 K is shown in Fig. S4(a).† Here, no additional (1 × 1) terraces and only few linear (1 × 2)-like defects are observed, and the precursor TiO_*x*_ point defects dominate. Overall, this strongly suggests that CO_2_ acts as a weak oxidizing agent even on the bare rutile TiO_2−*x*_ surface. As we observed no accumulation of carbon in STM or AES, the most likely mechanism is that CO_2_ reacts to CO, leaving oxygen to form new TiO_2_ with Ti_int_ from the bulk. Post-annealing in UHV at 800 K [STM image in Fig. S4(c)†] mostly removes the point defects in favour of (1 × 1) terraces and (1 × 2) line defects.

Interestingly, no reaction at all is seen with CO_2_ on LR-TiO_2_. Fig. 2(c) shows an STM image obtained after 30 minutes in 1 mbar CO_2_ at 600 K. We observe only defects that are also present on the pristine surface [Fig. 1(a)], and crucially, not a single one of the pronounced bright TiO_*x*_ precursor species. Since these are ubiquitous both on LR-TiO_2_ reacting with O_2_ [Fig. 2(a)] and on HR-TiO_2_ reacting with CO_2_ [Fig. 2(d) and Fig. S4(a, b)†], we conclude that no reaction takes place between CO_2_ and LR-TiO_2_ under the conditions investigated here.

Turning from oxidizing to reducing gasses, Fig. 2(e and f) shows STM images of the two substrates exposed to 1 mbar H_2_ at 600 K. The image of LR-TiO_2_ [Fig. 2(e)] was acquired in UHV at RT, after 90 minutes at the reaction conditions. Images acquired during H_2_ exposure were qualitatively equivalent, but more poorly resolved. The surface appears more defective than directly after UHV preparation or after reaction with CO_2_ or CO. Various small defects may correspond to V_O_ and/or surface OH, though unambiguous assignment is difficult. There are also some slightly larger features with greater apparent height, which resemble the ones assigned as TiO_*x*_ precursor species under oxidizing conditions [Fig. 2(a and d)]. However, on this surface, we never observed any (1 × 2)-like row structures or patches after reaction with H_2_.

Again, the effect of NAP gas exposure is much more pronounced on the HR-TiO_2_ sample. The STM image shown in Fig. 2(f) was acquired during 1 mbar H_2_ exposure at 600 K. The step edges appear considerably more frayed than typical for an as-prepared surface, and a network of bright features covers most of the surface. These bright patches are again formed from rows like the ones seen in the (1 × 2) surface reconstruction. This is easiest to demonstrate by post-annealing the surface in UHV, as shown in Fig. S5.† After post-annealing to 773 K [Fig. S5(a)†], we obtain a well-ordered crosslinked (1 × 2) phase covering almost the entire surface. Interestingly, there are also some additional small (<5 nm) islands with (1 × 1) termination. Annealing to higher temperatures [973 and 1100 K, shown in Fig. S5(b and c),† respectively] removes most of the reduced phase, though the surface still exhibits significantly more (1 × 2)-like row defects than before H_2_ exposure, even after 20 minutes of annealing at 1100 K.

We have also studied the reaction of HR-TiO_2_ with H_2_ as a function of temperature. STM images acquired in 1 mbar H_2_ while sequentially increasing the temperature are shown in Fig. S6.† No significant increase in the number of defects is observed up to 473 K. At 523 K, bright features start to form on the terraces in a manner very similar to that observed in O_2_ on LR-TiO_2_. Initially we see primarily point features, which later accumulate into (1 × 2)-like rows. Increasing the temperature to 573 K increases the rate of this process, with more and more rows forming until the surface is largely covered.

Finally, we investigated the reaction of TiO_2_(110) with CO. Fig. 2(g and h) shows STM images of LR-TiO_2_ and HR-TiO_2_, respectively, both acquired in CO at 600 K, ≈40 minutes after reaching that temperature. On LR-TiO_2_, the surface appears unchanged from the pristine state after preparation in UHV. On HR-TiO_2_, we observe a slightly higher concentration of reduced (1 × 2)-like row defects and their usual point precursors than on the as-prepared surface. Interestingly however, unlike with H_2_, the reaction with CO seemed to quickly produce a small number of defects as seen in Fig. 2(h), but this growth then saturated and stopped. While we could show additional defect growth with longer exposure times in H_2_ [shown in Fig. S6†], the area seen in Fig. 2(h) remained completely unchanged over 10 minutes of imaging in CO, and even for long exposures, no full coverage of a (1 × 2) phase as observed in hydrogen [Fig. 2(f)] could be obtained. Note that the images shown here were acquired in 0.1 mbar for the LR-TiO_2_, but in 1 mbar for the HR-TiO_2_. We also tested the influence of pressure on HR-TiO_2_, and found no qualitative difference between 0.1 and 1 mbar exposure.

### Reactivity of Pt nanoparticles on TiO_2_

**Fig. 3:**
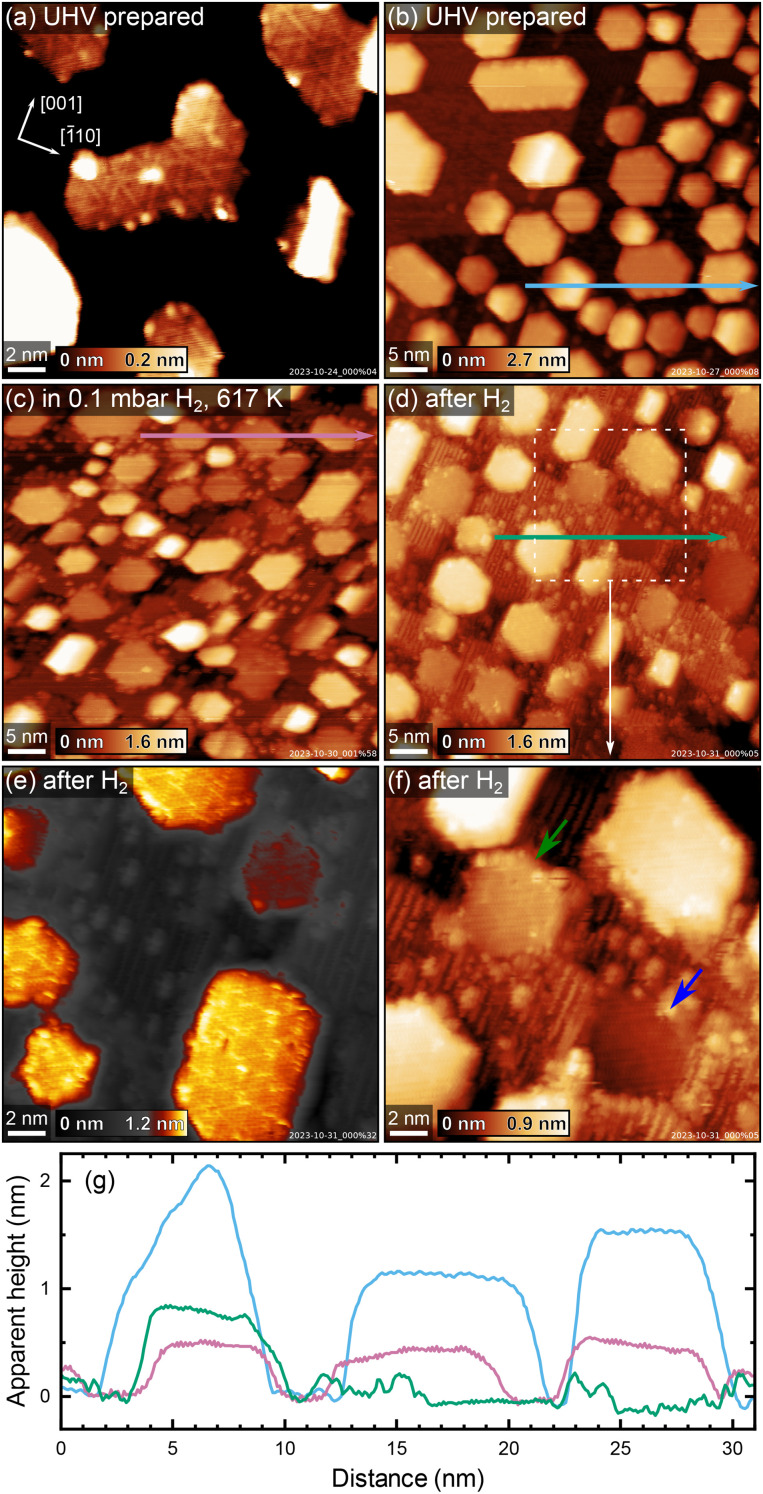
STM images showing the evolution of Pt nanoparticles on HR-TiO_2_ in H_2_. (a and b) As-sintered nanoparticles in UHV, (a) annealed 30 minutes at 500 K, 5 minutes at 1000 K and 30 minutes at 800 K, and (b) annealed another 30 minutes at 1200 K. (c) The same sample in 0.1 mbar H_2_ at 617 K, ≈84 minutes after a temperature of ≈600 K had been reached. (d–f) Images acquired after cooling to room temperature and returning the sample to UHV. Note the strongly amplified colour scale in (e). (f) Magnified view of the area marked with a dashed white square in (d). (g) Apparent height profiles measured along the horizontal arrows drawn in (b) (blue), (c) (pink), and (d) (green). Scanning parameters *U*_sample_ and *I*_tunnel_ were (a) 1.8 V, 0.1 nA, (b) 1.9 V, 0.4 nA, (c) 2.0 V, 0.7 nA, (d and f) 1.7 V, 0.2 nA, and (e) 2.5 V, 0.1 nA.

To investigate how the sample stoichiometry affects supported metal nanoparticles, we first deposited 7 monolayers (ML, defined as one atom per TiO_2_ unit cell, or 5.2 × 10^18^ m^−2^) of platinum on HR-TiO_2_. Initially, we tried sintering the particles by roughly following the recipe reported by Dulub *et al.*, annealing first at 500 K for 30 minutes, then at 1000 K for 5 minutes, and finally at 800 K for 30 minutes (200 °C, 700 °C and 500 °C, respectively were used by Dulub *et al.*).^16^ The Pt particles we obtained in this manner are more closely spaced and their lateral extension is much smaller than that reported previously, possibly due to the smaller amount of deposited material [7 ML *vs.* 25 ML (ref. 16)]. An STM image taken after the initial annealing treatment described above is shown in Fig. 3(a). Fig. 3(b) shows STM of the same sample after an additional 30 minutes of annealing in UHV at 1200 K. The particles are typically 5–10 nm wide, with a mean apparent height of 1.3 nm [see blue height profile in Fig. 3(g)]. We tried various different annealing treatments, as well as keeping the sample at 400 K during Pt deposition, but never obtained significantly larger particles than those shown in Fig. 3. Interestingly, we also observe that many particles are slightly tilted around the [001] axis with respect to the underlying TiO_2_(110) surface. When evaluating apparent particle heights, particles were therefore assigned the median value of their surface plane.

A superstructure indicative of encapsulation is resolved in STM already after the initial treatment in Fig. 3(a). Interestingly, the motif more closely resembles the “pinwheel” structure reported by Bowker *et al.* on Pd^18,19^ than the “zigzag” structure reported by Dulub *et al.* on Pt(111).^16^ Dulub *et al.* also reported an “iceberg” configuration of their particles with some Pt apparently embedded in the surface;^16^ in contrast, the overall area coverage and apparent height of our particles after UHV annealing are in good agreement with the nominal amount of deposited Pt, suggesting the nanoparticles are mostly located on top of the TiO_2_ surface.

After acquiring the data shown in Fig. 3(b), we exposed these particles to 0.1 mbar H_2_ at ≈600 K. A NAP-STM image acquired in hydrogen, ≈84 minutes after the temperature reached 600 K, is shown in Fig. 3(c). Note that the image appears somewhat distorted due to thermal drift of the STM scanner. Several changes can be observed on the surface. First, bright point defects and rows as seen on reduced TiO_2_(110)-(1 × 2) surfaces form on the substrate, similar to what was also observed on the bare surface [Fig. 2(f) and Fig. S6†]. Second, the substrate undergoes significant surface roughening, seen as an increased step density in between the Pt nanoparticles. Third, the apparent height of the Pt nanoparticles decreases with respect to their UHV-prepared state [compare the blue and pink height profiles in Fig. 3(g)], and some of them appear almost coplanar with the TiO_2_ substrate.

These changes are investigated more closely by the STM images in Fig. 3(d–f), which were acquired after cooling to room temperature and pumping to UHV. The mean apparent height of the encapsulated Pt particles in Fig. 3(d) is ≈0.5 nm [see green height profile in Fig. 3(g)], compared to ≈1.3 nm before hydrogen treatment (blue height profile), while the lateral extension of the particles remains largely unchanged. Note however that the mean apparent height in Fig. 3(d) comes with some systematic uncertainty, as it is difficult to clearly assign particles to a TiO_2_ terrace due to the increased number of step edges. Nevertheless, we can unambiguously say that the particles appear to be buried significantly in the surface after H_2_ treatment. This is especially clear when considering some particles in particular, which appear fully coplanar with a TiO_2_ terrace. Fig. 3(f) shows a magnified, contrast-adjusted view of the area marked by a dashed white square in Fig. 3(d). Here, the particle indicated by a green arrow appears roughly in-plane with the upper TiO_2_ terrace, while the particle indicated by a blue arrow clearly lies on the lower side of the step edge, in-plane with the lower TiO_2_ terrace (also seen in the green height profile in Fig. 3). Meanwhile, the superstructure observed before H_2_ treatment [Fig. 3(a)] appears unchanged, as seen in Fig. 3(e).

After the experiments shown in Fig. 3, we could again obtain a flatter and nearly defect-free surface by post-annealing in UHV at 1200 K for 30 minutes (STM image shown in Fig. S7†). Here, the mean apparent height of the Pt particles also somewhat increases again, from ≈0.5 nm to ≈0.7 nm.

We then performed the same series of experiments on LR-TiO_2_. We obtained a very similar size and shape distribution of particles as on HR-TiO_2_ after deposition of 7 ML Pt and sintering at 1000 K in UHV [Fig. S8(a)†]. While some nanoparticle surfaces seem to exhibit a poorly ordered internal structure [Fig. S8(d)†], we never clearly resolved an overlayer as seen on HR-TiO_2_, even after annealing at 1200 K in UHV. Unlike on HR-TiO_2_, the particles also appear largely unaffected upon exposure to 0.1 mbar H_2_ at 600 K [Fig. S8(b and e)†].

To confirm the absence of a TiO_*x*_ overlayer on UHV-annealed Pt nanoparticles on LR-TiO_2_, we performed C^18^O TPD measurements. Since these experiments took place in a different UHV setup, STM images of the as-prepared Pt particles on both supports were recorded in the TPD setup (Fig. S9†), which confirm that the surfaces closely resemble those obtained in the NAP-STM chamber on both HR-TiO_2_ [Fig. 3(b)] and LR-TiO_2_ [Fig. S8(a and d)†]. Fig. S10† shows XPS of the Pt 4f region, taken after Pt deposition and after subsequent sintering of the particles. While the peak shapes and areas for as-deposited Pt are almost identical, we observe a slightly higher binding energy (by ≈0.2 eV) of the Pt 4f peak on HR-TiO_2_ than on LR-TiO_2_ after sintering in UHV. This observation is consistent with a previously reported shift to higher binding energies upon particle encapsulation.^15^

**Fig. 4:**
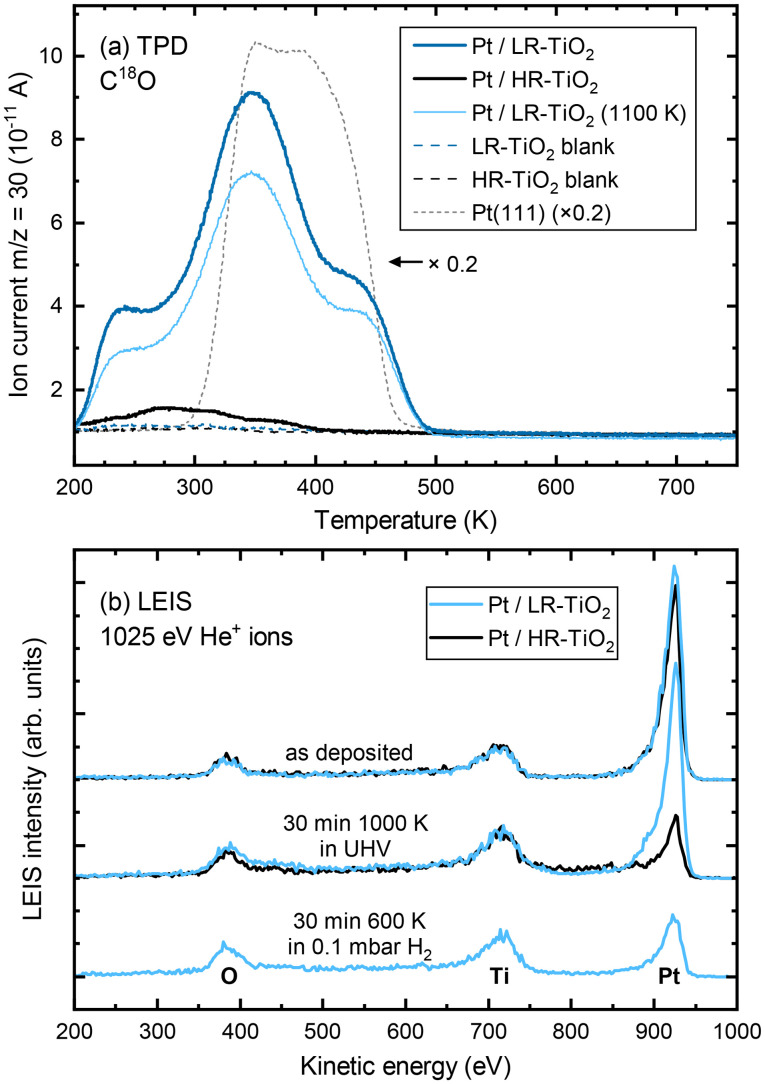
(a) C^18^O TPD (1 K s^−1^) of Pt nanoparticles on HR-TiO_2_ (black) and LR-TiO_2_ (blue). The dark blue and black curves were acquired after sintering Pt nanoparticles at 1000 K for 15 minutes on LR-TiO_2_ and HR-TiO_2_, respectively. The light blue curve was acquired after annealing the Pt/LR-TiO_2_ sample for an additional 75 minutes at 1100 K. Corresponding STM and XPS data are shown in Fig. S9 and S10, respectively. Black and blue dashed lines show blank measurements of the same samples before depositing Pt. A TPD curve from a Pt(111) single crystal is shown for comparison (scaled ×0.2, dashed grey line). (b) LEIS of Pt nanoparticles on HR-TiO_2_ (black) and LR-TiO_2_ (blue) directly after Pt deposition (top), after sintering at 1000 K in UHV (centre), and after exposing Pt/LR-TiO_2_ to 0.1 mbar H_2_ for 30 minutes at 600 K (bottom).

C^18^O TPD experiments of UHV-prepared Pt particles on both samples are shown in Fig. 4(a). Even at first glance, it is clear that the CO uptake on the two samples is markedly different, despite the very similar particle size and shape distributions seen in STM [Fig. S9(a and b)†]. Particles annealed at 1000 K on HR-TiO_2_ adsorb only a very small amount of CO compared to those on LR-TiO_2_ [compare the black and dark blue lines in Fig. 4(a), respectively]. Furthermore, while the main desorption features on Pt/LR-TiO_2_ appear at similar temperatures as on an extended Pt(111) surface, CO desorbs at a lower temperature on HR-TiO_2_. This is still the case even after further annealing the Pt nanoparticles on LR-TiO_2_ in UHV at 1100 K for 75 minutes. While the CO TPD trace [light blue line in Fig. 4(a)] is somewhat decreased compared to the one initially obtained on the same sample, it is still drastically higher than that on HR-TiO_2_, and the desorption temperatures of the peaks remain unchanged. Correlating TPD with STM, we can attribute the decrease in CO adsorption capacity between 1000 K and 1100 K annealing to further sintering of the particles [compare Fig. S9(b and c)†].

To further investigate this apparent difference in encapsulation behaviour, we also performed LEIS, as shown in Fig. 4(b). The as-deposited spectra show primarily platinum on both samples, though some oxygen and titanium signal remains. This indicates that for a deposition of 7 ML Pt, no closed Pt film is achieved, in good agreement with prior work.^15^ Next, the samples were annealed in UHV at 1000 K for 30 minutes. STM images taken after this annealing step are shown in Fig. S11.† At this point, LEIS shows a significant difference between the two samples [Fig. 4(b), centre]: while the peak ratios on LR-TiO_2_ are essentially unchanged, the Pt signal on HR-TiO_2_ is decreased significantly. This is in good agreement with the effect seen in TPD [Fig. 4(a)], also indicating encapsulation on the HR-TiO_2_, but not the LR-TiO_2_ sample. Corresponding XPS data is shown in Fig. S12.† Again, we see a slightly higher binding energy (by ≈0.2 eV) of the Pt 4f peak on HR-TiO_2_ than on LR-TiO_2_ after sintering in UHV.

Finally, we exposed the LR-TiO_2_ sample to 0.1 mbar H_2_ and heated it to 600 K while acquiring NAP-XPS (Fig. S13†). Interestingly, when the sample is kept at 600 K, the Pt 4f signal increases, while the O 1s and Ti 2p signals decrease. The original peak ratios are largely restored when the sample is returned to UHV and room temperature after 30 minutes of H_2_ exposure, aside from a very slight increase in the Ti^3+^ component of the Ti 2p peak. We tentatively assign this to a spreading of the particles due to a change in metal–support interaction, resulting in a slightly higher area coverage of Pt particles in the H_2_ atmosphere, possibly also visible in Fig. S8(b)† compared to Fig. S8(a and c).† LEIS data after this H_2_ exposure [Fig. 4(b), bottom] indicates that under these conditions, the particles on LR-TiO_2_ also become encapsulated, as the Pt signal is strongly decreased with respect to Ti and O, comparable to that on HR-TiO_2_ after UHV annealing.

## Discussion

Overall, the various differences in reactivity between the bare LR- and HR-TiO_2_ surfaces as well as that of supported Pt nanoparticles on those surfaces confirm the importance of controlling the rutile TiO_2_ sample stoichiometry. While the importance of support reduction state for *e.g.* the SMSI effect has been known for decades, unfortunately, much of the existing surface science literature relies on sputtering and “reoxidation” by UHV annealing. While this works fine for a limited number of cycles, it is inherently ill-defined, and leads to a creep in sample stoichiometry over time. It is easy to see that sample stoichiometry will converge for any recipe which includes a sufficiently oxidizing step to compensate the reduction from sputtering and high-temperature UHV annealing. Here, we have presented two recipes producing a consistent support stoichiometry, either highly reduced or near-stoichiometric. We have shown that these recipes provide equivalent results when two sets of TiO_2_ samples have been prepared in two different UHV systems.

### Interstitial diffusion

Since many of the experimental results rely on extracting Ti_int_ from the subsurface, it is interesting to consider how well the stoichiometry is equilibrated throughout the samples as a function of depth *z* (*i.e.* distance from the surface). Since the Ti_int_ are extremely dilute in the bulk (*x* < 4 × 10^−4^ in TiO_2−*x*_),^29^ their diffusion can be approximated by a one-dimensional random walk, where stepping along the symmetry-equivalent [100] and [010] directions each corresponds to a step by one layer towards or away from the (110) surface. The probability distribution resulting from such a random walk for any given Ti_int_ after *n* ≫ 1 steps is simply a normal distribution centred at its original position, with a root mean square distance given by
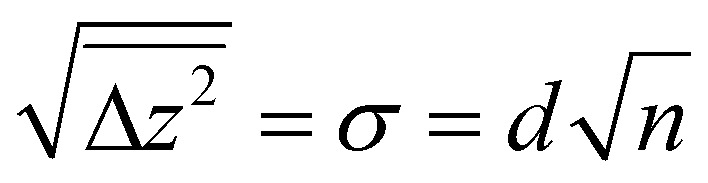
with step width *d* = 3.25 Å. The number of steps *n* after time *t* follows
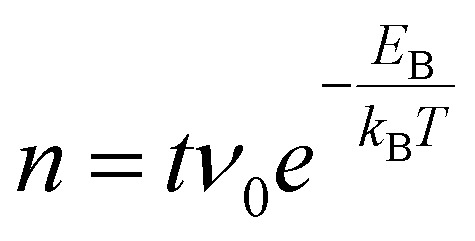
with a preexponential factor *ν*_0_ typically on the order of 10^13^ s^−1^. A surprising spread of values is reported in the literature for the activation barrier *E*_B_ of Ti_int_ bulk diffusion. While tracer diffusion and conductivity relaxation studies consistently report *E*_B_ ≈ 0.5 eV perpendicular to the [001] direction [*i.e.* towards or away from the (110) surface],^44–46^ contrasting values were found in STM-based studies. Smith *et al.* observed the growth of new TiO_2_ layers in low pressures of oxygen, and found a linear dependence on oxygen pressure and an apparent activation energy of only 0.25 eV.^36^ Since the process continued indefinitely without exhausting the subsurface Ti_int_, the authors concluded that the activation energy in the bulk must be of the same magnitude.^36,47^ In sharp contrast, Zhang *et al.* investigated the oxidation of single Ti_int_ atoms at the surface between 360 K and 400 K with STM and electron-stimulated desorption (ESD), and extrapolated an activation energy of *E*_B_ ≈ 1.0 eV.^37^ This was supported by DFT calculations, where a barrier of 1.2 eV was obtained for subsurface-to-surface diffusion, though interestingly the same work shows a lower barrier of 0.75 eV for diffusion to the subsurface from a deeper layer.^48^

Based on these considerations, we can use the standard deviations *σ* for Ti_int_ positions after a given number of steps as a measure of typical diffusion lengths when annealing. For example, assuming a diffusion barrier of 0.5 eV, *σ* is as large as 0.3 mm after only one minute of annealing at 900 K, and on the order of 0.1 mm after one minute even at 600 K. We can therefore expect rapid equilibration throughout the bulk for typical sample thicknesses of 0.5 to 3 mm, and high availability of Ti_int_ at the surface even at low bulk concentrations. Standard deviations *σ* as a function of the assumed bulk diffusion barrier are shown in Fig. S14(a)† for selected times and temperatures.

Separate from the experiments reported above, we have performed studies on 0.5 mm thick TiO_2_(110) samples, which we bulk-reduced by sputtering with 1 keV Ar^+^ ions while annealing at 1100 K for several hours until they exhibited a (1 × 2) LEED pattern. When exposing these samples to 0.1 mbar O_2_ at 600 K, we have found that they can be fully reoxidized (as judged by a change in colour from opaque black to transparent yellow-white) over the course of several hours. Assuming only a bulk diffusion barrier of 0.5 eV, one would expect a much faster reoxidation, on a timescale of only minutes. It is reasonable to assume that under these conditions, the availability of oxygen from the gas phase is not rate-limiting. Likewise, previous DFT calculations suggest that the O_2_ dissociation is barrierless once a Ti_int_ diffuses to the surface and interacts with an O_2_ molecule.^48^ These species must then diffuse to form new rutile terraces before the next layer can form.^36^ It seems plausible that this more complex, multi-step process has a higher effective barrier, which can explain the apparent discrepancy between reported bulk diffusion barriers^44–46^ and the ≈1 eV surface oxidation barrier obtained from ESD.^37^ Note that for bulk diffusion, we can rule out both the proposed 0.25 eV (ref. 37) and 1.0 eV (ref. 36) barriers, as they would result in much faster and much slower reoxidation, respectively.

**Fig. 5:**
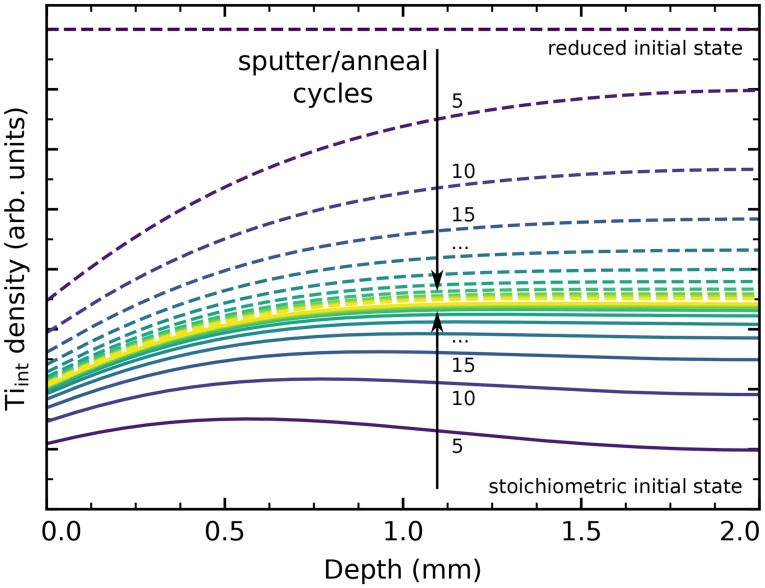
Ti_int_ concentration profiles in a 2 mm thick rutile TiO_2_(110) crystal from a simple diffusion simulation, shown after a given number of cycles of sputtering and annealing in O_2_ for 20 minutes at 900 K. Here, the bulk diffusion barrier was set to 0.6 eV, with a surface reaction barrier of 1 eV determining the rate of reoxidation. Solid and dashed lines show equilibration when starting from a fully stoichiometric and from a homogeneously reduced initial state, respectively, both arriving at a stationary state shown in yellow.

We have performed simple simulations (described in more detail in the ESI†) to model Ti_int_ diffusion following the random-walk scheme outlined above. This allows us to qualitatively compare our experimental results to the different barriers reported in the literature, and to estimate how many preparation cycles are required to equilibrate a sample. Fig. 5 shows depth profiles of the Ti_int_ distribution in a 2 mm thick sample resulting from cycles of sputtering (reducing the surface) and oxygen annealing at 900 K for 20 minutes, assuming barriers of 0.6 eV for bulk diffusion, 1.0 eV for oxidation at the surface (neglecting pressure dependence), and a preexponential factor *ν*_0_ = 10^13^ s^−1^. We choose to show the slightly higher (0.6 eV) than commonly reported barrier here because it more clearly illustrates the evolution of bulk concentration profiles. The same plot for a barrier of 0.5 eV is shown in Fig. S14(b).† Solid lines show the evolution of a sample starting from a fully oxidized state, while dashed lines start from an arbitrarily but homogeneously reduced state. In both cases, the sample is well-equilibrated after about 30 cycles. The remaining stoichiometry gradient at the surface is due to the continual reoxidation, and is less pronounced for a smaller bulk diffusion barrier, as shown in Fig. S14(b).† If we include our final annealing step of 1100 K in the simulation, and assume that no further oxidation takes place at that point due to the strongly reduced oxygen chemical potential *μ*_O_2__, then no stoichiometry gradient remains from the surface to the bulk, as shown in Fig. S14(c).† The bulk concentration always converges to a constant value in these simulations, which matches the experimental observation of a reproducible crystal stoichiometry (as judged by surface defects and the sample colour) over hundreds of cycles.

Overall, our results are generally consistent with a ≈0.5 eV barrier for bulk Ti_int_ diffusion perpendicular to the (110) surface, as reported in tracer diffusion studies.^44–46^ However, we conclude that a higher barrier for oxidative reaction of Ti_int_ at the surface should be assumed, as we otherwise would expect even faster reoxidation of heavily reduced samples than is actually observed. This is in good agreement with the work by Zhang *et al.*, who estimate a barrier of ≈1.0 eV for reaction with adsorbed oxygen below 400 K.^37^

### Oxidizing conditions

Qualitatively, the rapid growth of TiO_*x*_ ad-species in O_2_ and, ultimately, the formation of new TiO_2_ terraces is easy to understand. While the rate of material growth was observed to scale linearly with *p*_O_2__ at lower pressure [<10^−5^ mbar, orange lines in Fig. 1(c)],^36^ the impingement rate of O_2_ per TiO_2_(110) surface unit cell is 5.2 × 10^4^ s^−1^ at 600 K and 0.1 mbar. It therefore seems unlikely that the availability of O_2_ at the surface is still rate-limiting. As discussed above, assuming a bulk diffusion barrier for Ti_int_ of 0.5 eV, bringing titanium to the surface is likewise extremely fast. The rate of material growth is then mainly determined by an effective surface reaction barrier, which must include not only the reaction of Ti_int_ with O_2_ at the surface, but also the accumulation and structural rearrangement of new TiO_*x*_ precursor material into bulk-structured TiO_2_ terraces. The previously reported value of ≈1.0 eV (ref. 37) should therefore be seen as a lower bound. The results on LR-TiO_2_ indicate that at 600 K, on-surface diffusion of the precursor point defects is sluggish, and once formed, terraces remain small rather than accumulating into larger ones. Indeed, on HR-TiO_2_, LEED and (after post-annealing) STM both indicate highly disordered growth and surface roughening. The observation of bulk defects (seen as screw dislocations and half-steps in STM, data not shown) after NAP oxygen exposure also fits into this picture. The much higher rate of growth on HR-TiO_2_ is readily explained by the higher availability of Ti_int_ in that sample.

In contrast to O_2_, we observe a clear qualitative difference between the LR-TiO_2_ and HR-TiO_2_ samples in CO_2_. While the NAP exposure is clearly mildly oxidizing on HR-TiO_2_, no material growth at all is observed on LR-TiO_2_. Though one may reasonably expect a slower rate of oxidation based on the lower availability of Ti_int_, comparison with the NAP O_2_ experiment still suggests that TiO_*x*_ species should also form on LR-TiO_2_, albeit at a lower rate. The fact that not a single defect was found in STM even after 30 minutes at 600 K suggests that the reaction of Ti_int_ either has a significantly higher activation barrier on LR-TiO_2_ than on HR-TiO_2_, or is thermodynamically unfavourable.

Based on the fact that no carbon residue is found on the surface by AES and STM after oxidation with CO_2_, we can assume that CO_2_ reacts to CO, providing an oxygen to bind to Ti_int_ diffusing from the bulk. The full reaction can then be written asTi_int_^3+^ + 2CO_2_(g) + 3e^−^ → TiO_2_(s) + 2CO(g).

While the exact reaction process is likely to be complex, with multiple CO_2_ molecules interacting with an active site, we can at least estimate the overall reaction enthalpy Δ*H* of such a process. The reaction of O_2_ with Ti_int_ has been investigated by DFT in previous work, where the authors found an energy gain of 6.6 eV from forming an on-surface TiO_2_ ad-species, compared to Ti_int_ in a subsurface layer and O_2_ in the gas phase.^48^ The cost of reducing CO_2_ to CO, CO_2_(g) → CO(g) + ½O_2_(g), is well-known (Δ*H*_0_ = 2.93 eV).^41^ Comparing directly, we obtain an energy gain of ≈0.7 eV for oxidation of Ti_int_ to a TiO_2_ ad-species, using two CO_2_ molecules from the gas phase.

We speculate that the surprising inertness of the LR-TiO_2_ surface is likely related to the different electronic structure in comparison to HR-TiO_2_. Activating adsorbed CO_2_ typically requires first transferring an electron to the molecule.^49^ Initiating the reaction with Ti_int_ thus requires two charge transfer events to adsorbed molecules in close proximity. This is in contrast to the reaction with oxygen, which can proceed in a single step through reaction of Ti_int_ with an O_2_ molecule.^48^ Rutile TiO_2−*x*_ is an n-type semiconductor, with the Fermi level position (relative to the band structure) determined by the doping level, which in our samples corresponds simply to the Ti_int_ concentration. The stoichiometry of the crystal thus determines not only the number of available charge carriers, but also their energy relative to adsorbed species. It therefore seems plausible that on the LR-TiO_2_ sample, the reaction with CO_2_ may be completely inhibited, rather than just proceeding at a lower rate than with O_2_.

### Reducing conditions

In reducing atmospheres, we observe that both H_2_ and CO further reduce HR-TiO_2_, though the initial reaction with CO quickly stops. Intuitively, it makes sense that reducing the surface should form the (1 × 2) phase, which is generally assigned as Ti_2_O_3_ (though the crosslinked precursors may be less reduced).^32,40^ However, the atomistic mechanism is less clear than for oxidation. It seems that new (1 × 2)-like features form as ad-species on each terrace, nucleating seemingly at random. This begs the question where the material for these defects is drawn from.

Bowker and Bennett have investigated the thermal reduction process of a (1 × 2)-terminated TiO_2_(110) sample in UHV, and observed material loss in the form of retraction of (1 × 2) steps, without interconversion to (1 × 1).^47^ In that case, it is easy to conclude that oxygen is lost to the gas phase, and excess Ti diffuses to the bulk as Ti_int_. It is reasonable to assume that oxygen is similarly mainly removed from steps in the reaction with hydrogen, and indeed, the step edges often have a frayed appearance [see Fig. 2(f)]. Leftover Ti may then diffuse laterally to form the reduced surface phases as easily as moving to the bulk. However, forming Ti_2_O_3_ ad-features in this way clearly also requires oxygen, which must either be extracted from the step as well, or else from the underlying terrace.

On other reducible oxide surfaces like Fe_3_O_4_(001), accumulation of oxygen vacancies on a terrace typically results in the appearance of isolated, extended holes in the surface as excess Fe diffuses to the bulk.^50,51^ While hard to rule out entirely, we have not observed this on TiO_2_. An alternative mechanism may be that OH groups are formed at step edges, and that these diffuse on the surface either independently, or in combination with excess Ti from the same step edge as a Ti(OH)_*x*_ species. Such a precursor species could then react to Ti_2_O_3_ (or possibly a hydroxylated form thereof) on the surface. However, as the formation of ad-species close to steps is observed just as much as on terraces, nucleation may equally involve some additional terrace defect.

Post-annealing of HR-TiO_2_ in UHV leads to ordering of the reduced ad-features [Fig. S5(a)†] at low temperatures, and partly recovers a (1 × 1) surface at higher temperatures [Fig. S5(b and c)†]. This is easily understood as the same process seen in the “reoxidation” of samples by UHV annealing after sputtering, where excess Ti is dissolved into the bulk as Ti_int_. However, the concentration of (1 × 2) rows on the surface after post-annealing is still noticeably higher than before H_2_ exposure, indicating significant overall reduction of the sample.

It is interesting to note that no extensive reduction takes place in CO on either sample. The fact that the reaction stops after only a few reduced ad-species have been formed suggests that CO reacts solely with some preexisting defect, thus self-limiting the process. The observation of continued reduction of HR-TiO_2_ with hydrogen, but not with CO, suggests that the rate-limiting step is the extraction of lattice oxygen, rather than the adsorption and dissociation of H_2_. The inert behaviour of LR-TiO_2_ to CO exposure may indicate that the required defects are not available on that sample.

### Nanoparticle encapsulation

Concerning Pt nanoparticles, we very clearly see encapsulation on HR-TiO_2_ in STM (Fig. 3), TPD and LEIS (Fig. 4). In contrast, no overlayer was observed in STM on LR-TiO_2_ (Fig. S8†), and the substantial difference in CO uptake capacity between the two samples seen in TPD convinces us that encapsulation in UHV is inhibited on the near-stoichiometric sample. This is in good agreement with literature: it has long been known that reducing TiO_2_ is a prerequisite for the SMSI effect,^13,27^ and lower CO uptake capacity was already reported in the original work on SMSI by Tauster.^13,14^ However, exposing the sample to hydrogen under near-ambient pressure conditions at 600 K finally results in encapsulation also on LR-TiO_2_ as indicated by LEIS [Fig. 4(b)], likely due to surface reduction.

Our findings are in good agreement with previous reports that encapsulation mainly depends on the doping level of the support, rather than the availability of excess Ti.^20^ Based on the material growth of the bare samples in a NAP oxygen atmosphere, we can conclude that Ti_int_ can easily diffuse to the surface even at 600 K, including on samples that do not exhibit an SMSI effect. The different interaction of the samples with Pt must therefore be thermodynamically preferred, rather than dictated by kinetic limitations.

Exposing the Pt particles to 0.1 mbar H_2_ at 600 K did not result in any significant changes to particle size distributions, or to the nature of an existing TiO_*x*_ overlayer. Unexpectedly however, on HR-TiO_2_, we observe a significant decrease in apparent height of the particles. Based on the concomitant roughening of the support, we speculate that TiO_2_ material from underneath the particles diffuses to form new terraces covering their side facets. This partial burying of the particles is an interesting effect which would hardly be seen by area-averaging techniques like XPS or TPD, where side facets of these relatively flat particles do not contribute strongly. However, it is likely not particularly significant to catalyst activity, as the particle overlayer remains the same. As to what constitutes the driving force for particles becoming embedded in the surface, two explanations are possible: first, that interaction with hydrogen increases the surface energy of side facets covered with the TiO_*x*_ suboxide overlayer beyond the interface energy of side facets embedded in stoichiometric TiO_2_; or second, that an embedded configuration is already thermodynamically favoured when the particles are first overgrown, but cannot be accomplished due to kinetic limitations. In the second model, the intense surface roughening and restructuring by hydrogen also seen on the bare TiO_2_(110) surface [Fig. 2(f)] would allow moving the large particles into a more favourable position. However, taking into account the slightly recovered mean apparent particle heights after UHV post-annealing (Fig. S7†), a modified thermodynamic equilibrium in H_2_ atmosphere appears more plausible. It is interesting to note that significant material transport within the TiO_2_ substrate can clearly take place even at 600 K to accommodate this.

## Conclusions

In conclusion, we have found significant differences in surface reactivity of a near-stoichiometric (lightly reduced, LR-TiO_2_) and a highly reduced (HR-TiO_2_) TiO_2_(110) sample. Near-ambient pressure atmospheres of O_2_, CO_2_, H_2_ and CO at 600 K all induced surface oxidation or reduction on the bare HR-TiO_2_ sample, while LR-TiO_2_ was only mildly oxidized by O_2_ and appears completely inert to the other three gasses. Similarly, Pt particles were encapsulated already after UHV sintering on HR-TiO_2_, while encapsulation on LR-TiO_2_ was inhibited in UHV, and only occurred when the sample was exposed to 0.1 mbar of H_2_ at 600 K. Surprisingly, the already encapsulated particles on HR-TiO_2_ were further modified in the hydrogen environment, where they were embedded deeper into the TiO_2_ surface. Control of the sample stoichiometry over many preparation cycles has proven crucial in investigating these differences. The recipes we have presented for the preparation of TiO_2_(110) samples with reproducible bulk stoichiometry will enable a much more controlled surface preparation and thus help prevent discrepancies and controversies in future experiments.

## Author contributions

FK: conceptualization, investigation, data curation, formal analysis, visualization, writing – original draft; MK: conceptualization, investigation, data curation, formal analysis; SK: investigation, formal analysis, visualization; JR: investigation; AJ: investigation, formal analysis; MF: investigation; GA: investigation; FE: resources, supervision; BAJL: conceptualization, funding acquisition, resources, supervision, project administration; all authors: writing – review & editing.

## Data availability

The data supporting this article have been included as part of the ESI.†

## Conflicts of interest

There are no conflicts to declare.

## Supplementary Material

NR-016-D4NR01702A-s001

NR-016-D4NR01702A-s002

NR-016-D4NR01702A-s003
