# Supplementary material for: The influence of bulk stoichiometry on near-ambient pressure reactivity of bare and Pt-loaded rutile TiO2(110)
Source: Nanoscale. 2024 Aug 29;16(38):17825–37. doi: 10.1039/d4nr01702a (PMC11381967; doi:10.1039/d4nr01702a)
Supplement: NR-016-D4NR01702A-s001 [file NR-016-D4NR01702A-s001.pdf]

Supplementary Information (SI) for Nanoscale.
This journal is © The Royal Society of Chemistry 2024

The influence of bulk stoichiometry on near-ambient pressure reactivity of bare and Pt-loaded rutile TiO_2(110)

Florian Kraushofer,[a,†] Matthias Krinninger,[a,†] Sebastian Kaiser,[a,b] Johanna Reich,[a] Agnieszka Jarosz,[a] Matthias Füchsl,[a] Gaurav Anand,[a] Friedrich Esch[b] and Barbara A.J. Lechner* [a,c]

[a] Functional Nanomaterials Group & Catalysis Research Center, Department of Chemistry, TUM School of Natural Sciences, Technical University of Munich, Lichtenbergstr. 4, 85748 Garching, Germany

[b] Chair of Physical Chemistry & Catalysis Research Center, Department of Chemistry, TUM School of Natural Sciences, Technical University of Munich, Lichtenbergstr. 4, 85748 Garching, Germany

[c] Institute for Advanced Study, Technical University of Munich, Lichtenbergstr. 2a, 85748 Garching, Germany

* bajlechner@tum.de

† These authors contributed equally.

Supplementary Figures

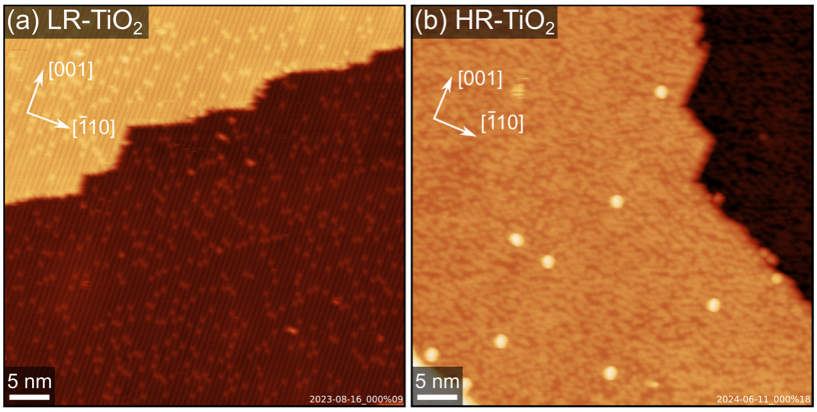

Figure S1. Additional larger-area STM images (50×50 nm^2) of as-prepared rutile TiO_2(110) surfaces, showing (a) the LR-TiO_2 sample (U_{sample} = 1.6 V, I_{tunnel} = 0.3 nA) and (b) the HR-TiO_2 sample (U_{sample} = 1.6 V, I_{tunnel} = 0.1 nA).

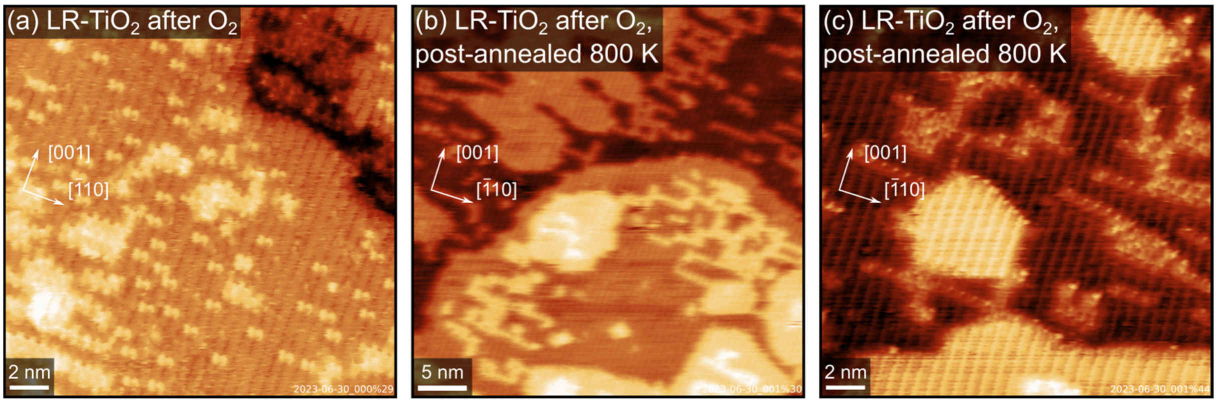

Figure S2. Additional STM images after NAP O_2 exposure of LR-TiO_2, from the same experiment as the image shown in Figure 2(a). (a) LR-TiO_2 after exposure to 0.1 mbar O_2 at 600 K for 15 minutes, and (b,c) after post-annealing at 800 K for 10 minutes in UHV. Images were acquired at RT in UHV, with scanning parameters U_{sample} and I_{tunnel} (a) 1.7 V, 0.1 nA, (b) 1.7 V, 0.2 nA and (c) 1.9 V, 0.1 nA.

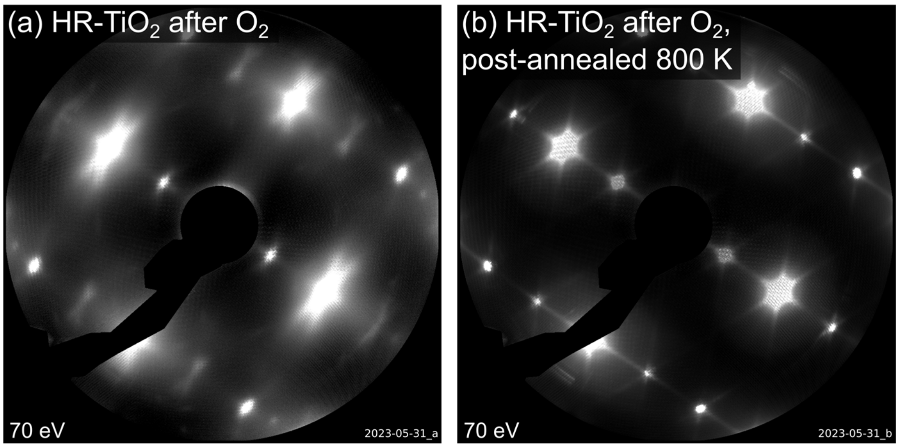

Figure S3. LEED (70 eV incident electron energy) of HR-TiO_2 (a) directly after exposure to 0.1 mbar O_2 at 600 K for 15 minutes [also shown as the inset in Figure 2(b)], and (b) after post-annealing at 1100 K for 10 minutes in UHV, corresponding to the STM image shown in Figure 2(b).

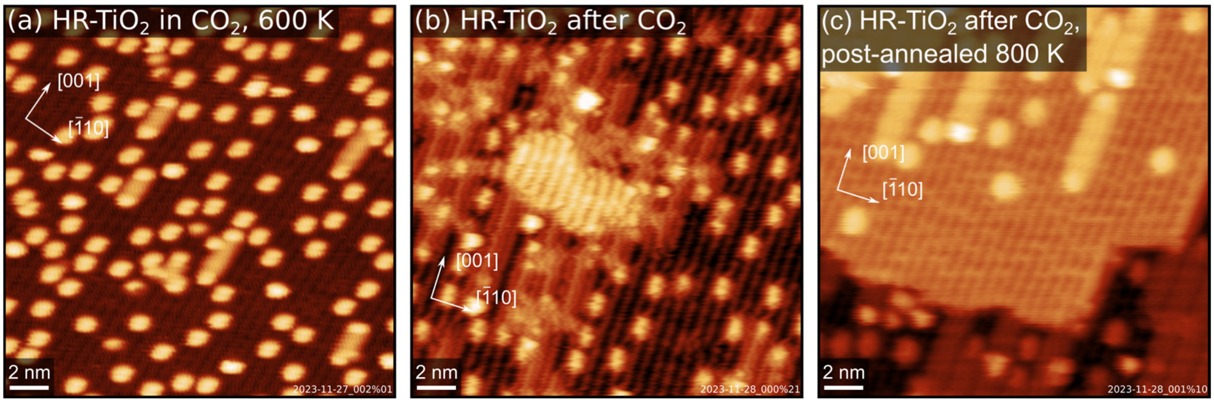

Figure S4. Additional STM images from the NAP CO_2 experiment on HR-TiO_2 shown in Figure 2(d). (a) HR-TiO_2 in 1 mbar CO_2, image acquired in gas atmosphere 10 minutes after reaching 600 K. (b) After exposure to 1 mbar CO_2 at 600 K for 30 minutes, image acquired at RT in UHV, same measurement as the image shown in Figure 2(d). (c) After post-annealing at 800 K for 10 minutes in UHV. Scanning parameters U_{sample} and I_{tunnel} were (a) 1.8 V, 0.2 nA, (b) 2.1 V, 0.2 nA and (c) 1.7 V, 3.3 nA.

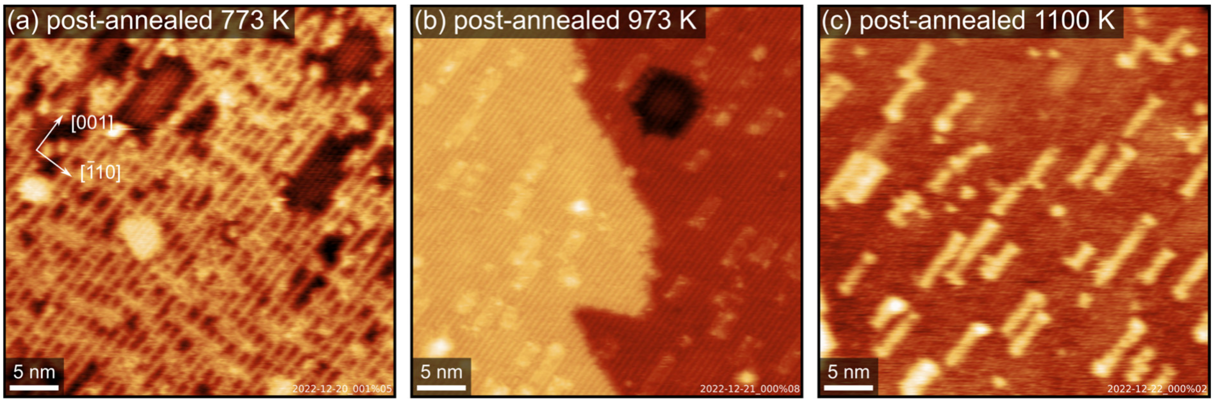

Figure S5. Additional STM images corresponding to the NAP H_2 experiment shown in Figure 2(f), showing the HR-TiO_2 sample post-annealed in UHV after exposure to 1 mbar H_2 at 600 K for 105 minutes. The sample was annealed at (a) 773 K for 10 minutes, (b) 973 K for 10 minutes, and (c) 1100 K for 20 minutes. Images were acquired at RT in UHV, with scanning parameters U_{sample} and I_{tunnel} (a,b) 1.2 V, 0.1 nA and (c) 1.2 V, 0.2 nA.

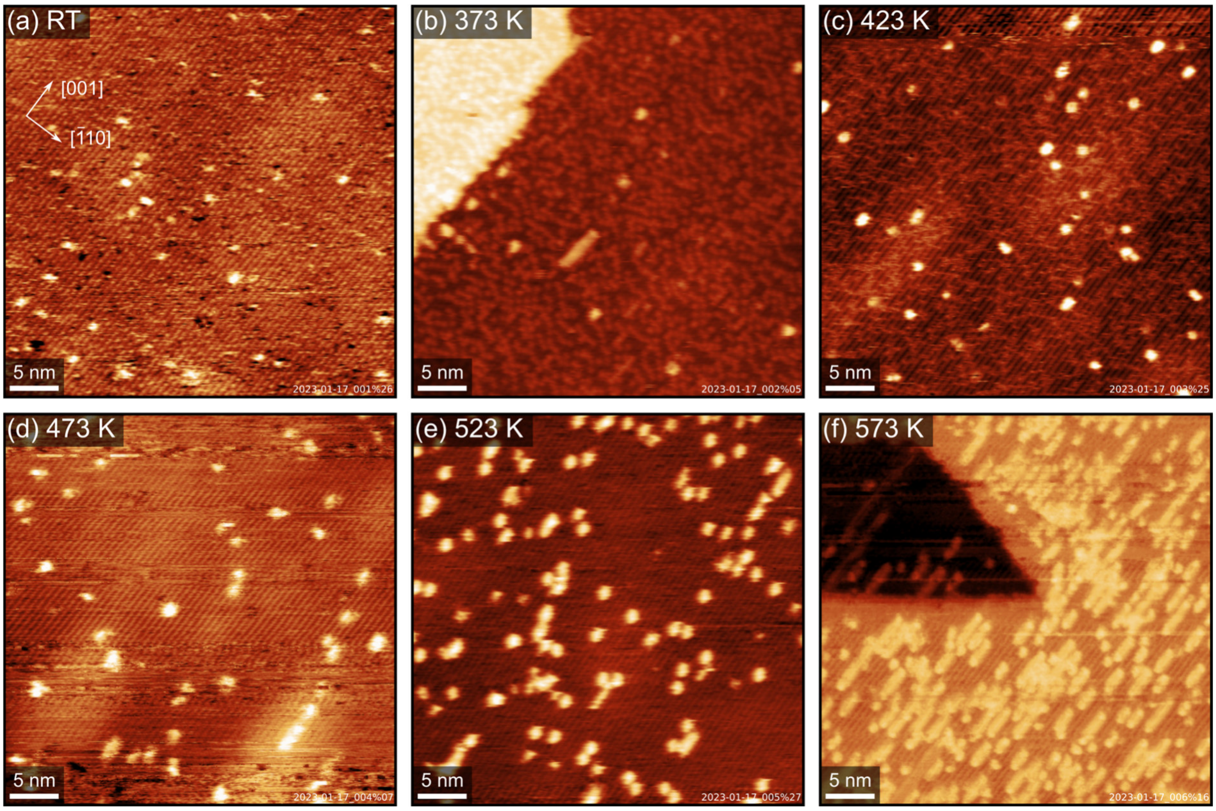

Figure S6. STM images of HR-TiO_2 in 1 mbar H_2 while heating to different temperatures. (a) RT; (b) 373 K, image acquired 7 minutes after reaching the temperature; (c) 423 K, image acquired after 27 minutes; (d) 473 K, image acquired after 13 minutes; (e) 523 K, image acquired after 21 minutes; (f) 573 K, image acquired after 74 minutes. Brightness modulations of the surface in (a), (c), (d) and (e) are due to Ar bubbles, which are typical for this surface after UHV preparation. Scanning parameters U_{sample} and I_{tunnel} were (a) 1.7 V, 0.2 nA, (b) 1.4 V, 0.1 nA, (c) 2.1 V, 0.2 nA, (d) 1.3 V, 1.0 nA, (e) 1.3 V, 0.9 nA and (f) 1.5 V, 0.7 nA.

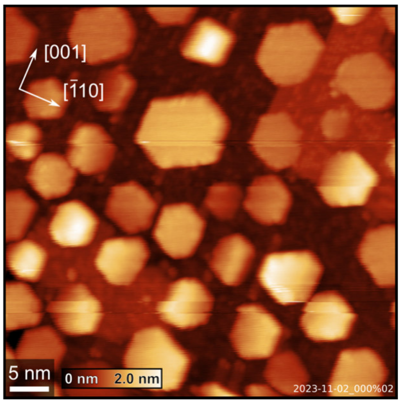

Figure S7. STM image showing Pt nanoparticles on HR-TiO_2, post-annealed at 1200 K in UHV for 30 minutes after the H_2 experiment shown in Figure 3. U_{sample} = 1.8 V, I_{tunnel} = 0.1 nA.

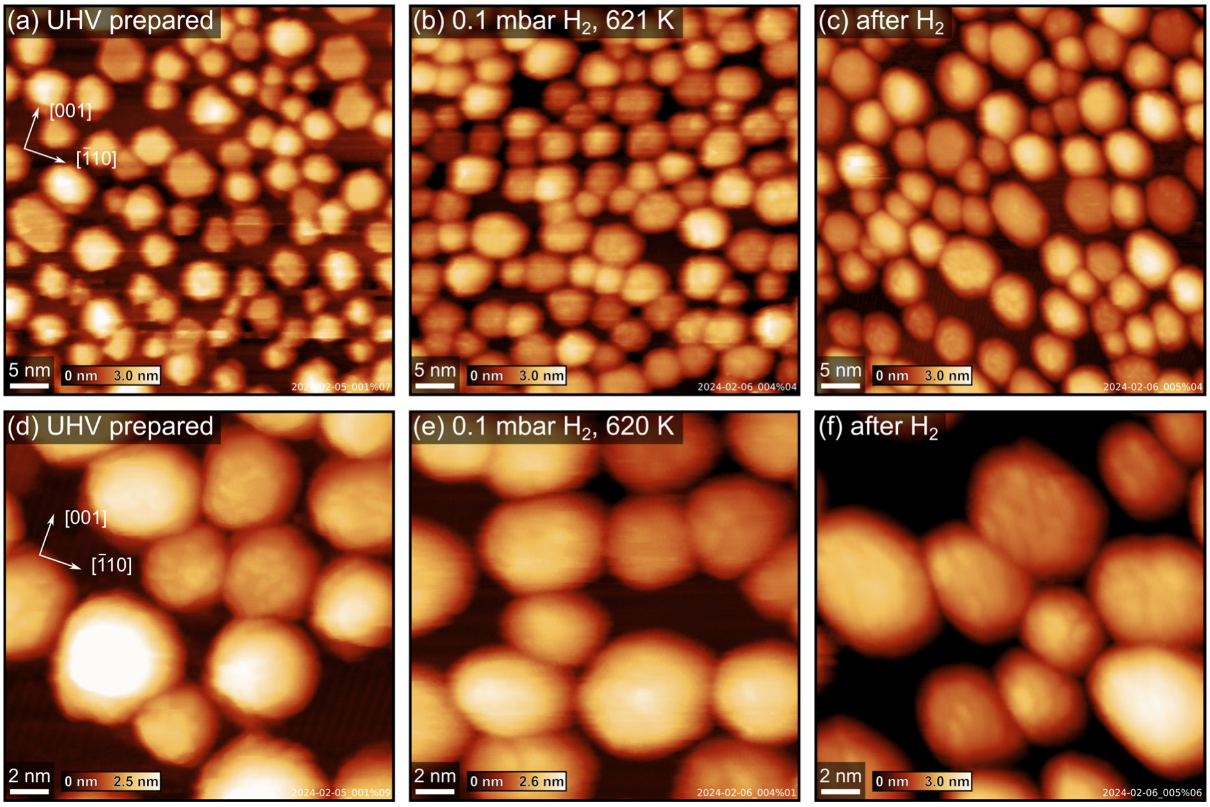

Figure S8. STM images showing the evolution of Pt nanoparticle on LR-TiO_2 in H_2. (a,d) As-sintered nanoparticles in UHV, annealed 30 minutes at 1000 K. (b,e) The same particles in 0.1 mbar H_2 at (e) 620 K, ≈15 minutes after the temperature reached 600 K and (b) 621 K, after ≈21 minutes. (c,f) Images acquired after cooling to room temperature and returning the sample to UHV after a total of 90 minutes at T ≥ 600 K in H_2. Some internal structure is resolved on some of the particles, but we did not find any well-defined superstructure. Scanning parameters U_{sample} and I_{tunnel} were (a) 1.4 V, 2.0 nA, (b) 1.4 V, 1.5 nA, (c) 1.2 V, 0.3 nA, (d) 1.4 V, 0.2 nA, (e) 1.4 V, 1.4 nA, (f) 1.2 V, 0.8 nA.

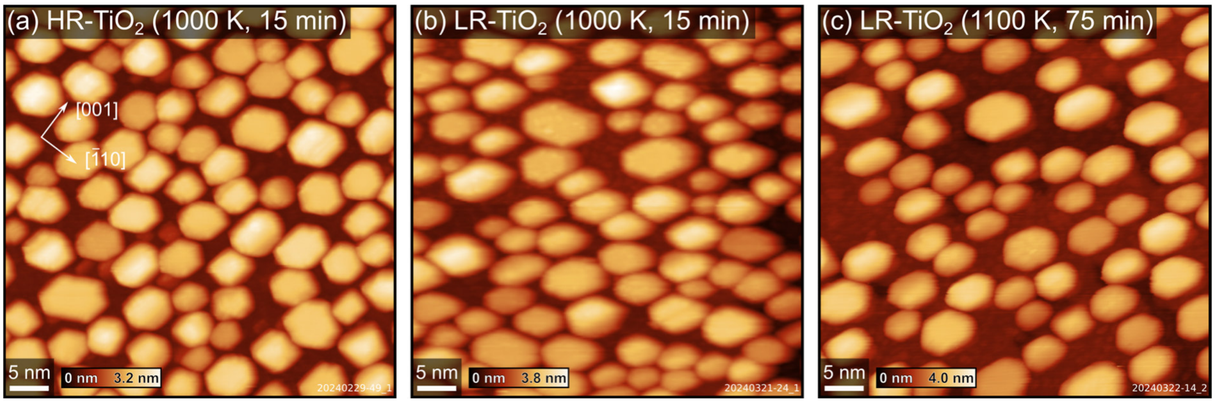

Figure S9. STM images of the Pt/LR-TiO_2 and Pt/HR-TiO_2 samples used in the $C^{18}O$ TPD experiments shown in Figure 4(a). (a) Pt particles on HR-TiO_2 after annealing in UHV at 1000 K for 15 minutes. (b) Pt particles on LR-TiO_2 after annealing in UHV at 1000 K for 15 minutes, and (c) after annealing in UHV at 1100 K for 75 minutes. Slight linear distortions in (b) and (c) are due to thermal drift of the STM scanner. Scanning parameters U_{sample} and I_{tunnel} were (a) 1.5 V, 1.0 nA and (b, c) 2.0 V, 0.3 nA.

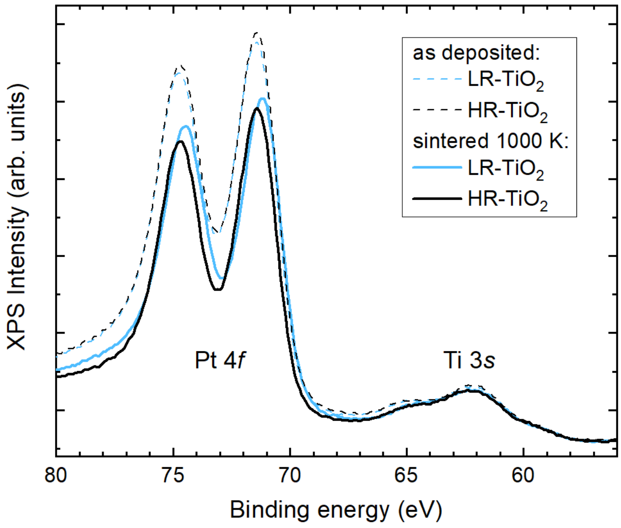

Figure S10. XPS (Al Kα, normal emission, 50 eV pass energy) of the Pt/LR-TiO_2 and Pt/HR-TiO_2 samples used in the $C^{18}O$ TPD experiments shown in Figure 4(a). Dashed lines show spectra acquired directly after depositing Pt. Solid lines show spectra acquired after annealing at 1000 K for 15 minutes. Note that the signal in the Ti 3*s* region is convoluted with duplicates of the Pt 4*f* peaks due to X-ray satellites from the non-monochromatic Al Kα source (α_3: ΔE = 9.8 eV, α_4: ΔE = 11.8 eV relative to $\alpha_{1,2}$, with relative intensities of 6.4% and 3.2%, respectively).[1]

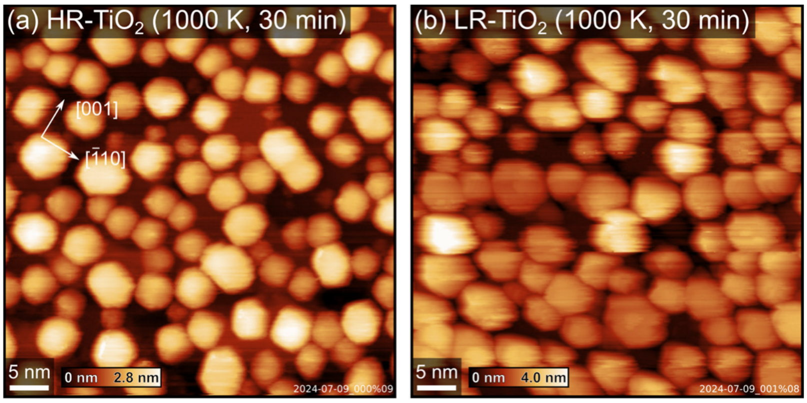

Figure S11. STM images of Pt/LR-TiO_2 and Pt/HR-TiO_2 corresponding to the LEIS data shown in Figure 4(b). (a) Pt particles on HR-TiO_2 after annealing in UHV at 1000 K for 30 minutes. (b) Pt particles on LR-TiO_2 after annealing in UHV at 1000 K for 30 minutes. Scanning parameters U_{sample} and I_{tunnel} were (a) 1.5 V, 1.2 nA and (b) 1.5 V, 0.9 nA.

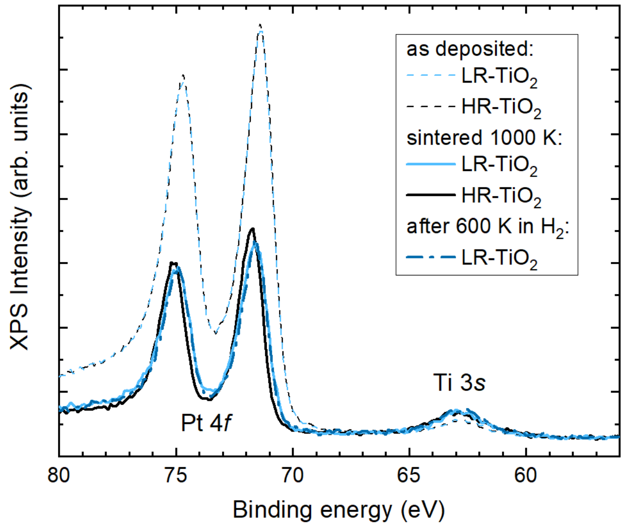

Figure S12. XPS (monochromated Al Kα, normal emission, 30 eV pass energy) of Pt/LR-TiO_2 and Pt/HR-TiO_2 corresponding to the LEIS data shown in Figure 4(b). Dashed lines show spectra acquired directly after depositing Pt. Solid lines show spectra acquired after annealing at 1000 K for 30 minutes. The dot-dashed, dark blue line was taken after exposing the LR-TiO_2 to 0.1 mbar H_2 and heating to 600 K for 30 minutes. Note the more pronounced difference between as-deposited and sintered Pt than seen in Figure S10, possibly indicating lower Pt loading in the TPD experiment.

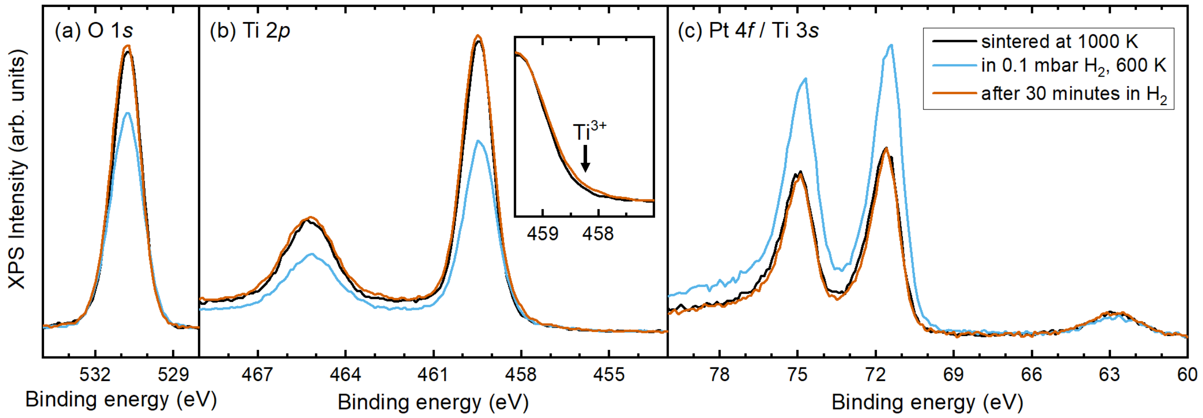

Figure S13. NAP-XPS (monochromated Al Kα, normal emission, 30 eV pass energy) of the (a) O 1s, (b) Ti 2p and (c) Pt 4f / Ti 3s regions of Pt/LR-TiO_2 exposed to 0.1 mbar H_2 at 600 K. The spectra before (black) and after (orange) H_2 exposure were acquired in UHV at room temperature, while the blue curve was acquired in 0.1 mbar H_2 at 600 K. The inset in panel (b) is a magnified view of the Ti 2$p_{3/2}$ peak, indicating a slight increase in the Ti^{3+} component after H_2 exposure. The black and orange curves in panel (c) are the same as shown in Figure S12 and correspond to the LEIS data shown in Figure 4(b).

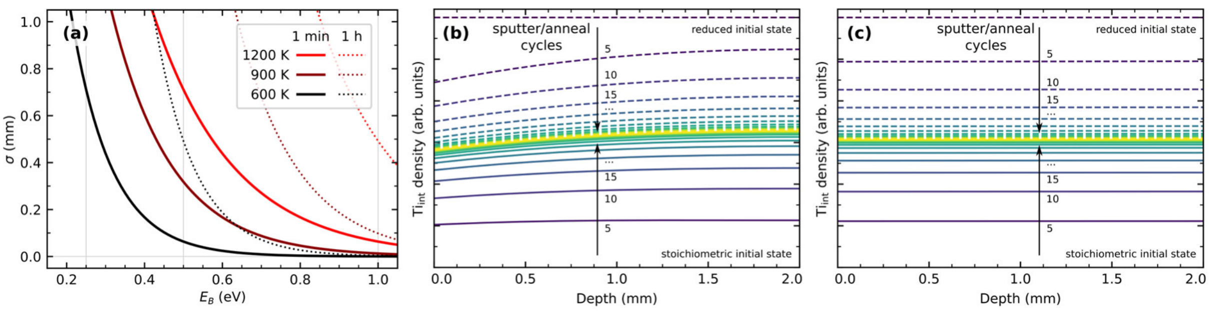

Figure S14. (a) Standard deviation σ for the position of a Ti_{int} interstitial diffusing perpendicular to the (110) surface in the bulk of rutile TiO_2(110) as a function of the activation barrier for bulk diffusion, shown for relevant temperatures and times. The interstitial is assumed to follow a one-dimensional random walk, resulting in a normal distribution with σ equivalent to the root mean square distance from the original position. (b) Simulated concentration profiles as shown in Figure 5 for a 2 mm thick rutile TiO_2(110) crystal after a given number of cycles of sputtering and annealing in O_2 for 20 minutes at 900 K. In contrast to Figure 5, here, the bulk diffusion barrier was set to 0.5 eV. The surface reaction barrier determining the rate of reoxidation was kept at 1 eV. Solid and dashed lines show equilibration when starting from a fully stoichiometric and from a homogeneously reduced initial state, respectively. (c) Same simulation as in (b), adding a 10 minute annealing step at 1100 K in each cycle. No further oxidation or reduction is assumed during this higher-temperature step.

Diffusion simulations

As discussed in the main manuscript, diffusion simulations of Ti_{int} were based on a one-dimensional random walk. This is a reasonable approximation when the diffusing particles are dilute enough that interaction between them is negligible. We further model the occupation of each layer as a floating-point concentration, rather than an integer number of diffusing particles. For each single Ti_{int}, the probability of finding it at a distance *z* from its original position is given by a normal distribution, with $\sqrt{\overline{\Delta z^2}} = \sigma = d\sqrt{n}$ after *n* steps, where *n* depends on the elapsed time, the diffusion barrier and temperature, and a preexponential factor (see main manuscript). We can then directly evaluate the concentration profile of a sample after a given annealing step by convolution of the initial concentration per layer with a normal distribution, setting the standard deviation σ to reflect annealing time and temperature. This effectively smears out each "particle" in the original concentration profile to reflect its likely position after annealing. Crucially, the result is exactly the same no matter if the concentration profile samples each atomic layer individually, or only every k^{th} layer, as long as σ is chosen according to the actual layer thickness. This treatment is therefore extremely computationally efficient, as only one calculation is required for each annealing step.

Edges of the sample must be accounted for specifically. The simplest boundary condition to implement is that when a particle at the surface (the 0th layer) would diffuse out of the surface (to the -1st layer), it is instead considered to still be in the 0th layer. This is easily achieved by performing the convolution with the normal distribution, then "folding back" the negative space, such that all concentration in the -1st layer is added to the 0th layer, all concentration in the -2nd layer is added to the 1st layer, and so on. It is easy to see that this is still an exact solution, as any diffusion event from the -1st layer is treated the same way as a diffusion event from the 0th layer, with diffusion in one direction having no effect, and diffusion in the other direction leading away from the surface. Applying the same approach to the other edge of the crystal, we essentially obtain periodic boundary conditions, where the concentration is flipped in every other period. Again, this is still an exact solution within the bounds of the random walk approximation, no matter the point sampling density.

Modelling oxidation at the surface is more difficult. To avoid having to model a varying thickness of the sample, we approximate oxidation by some probability that every time a particle would diffuse out of the surface, it disappears instead of staying in the surface layer. It is trivial to choose this probability to

correspond to some surface reaction barrier by setting it to a Boltzmann factor $e^{-\varepsilon/k_BT}$, with ε being the difference between the bulk and surface barriers.

Ideally, we would implement this oxidation process in our model by applying that probability every time a particle passes through the surface, *i.e.* from the 0th to the -1st layer or vice versa. However, the approach of simply convolving a normal distribution then breaks down, as *e.g.* most atoms at the 0th layer that would remain at the 0th layer in the random walk approximation have actually passed through the origin at least once, and likely many times (assuming large *n*). This can be solved either by calculating the contributions of different paths to each point of the normal distribution and applying the loss probability accordingly, or by choosing small time steps, such that few atoms diffuse out of the surface in each step. Both approaches are computationally expensive. We have chosen the second, applying the loss factor twice in each time step to the concentration in the out-of-surface space to account for the symmetrical nature of the problem (*i.e.*, for each particle found in the -1st layer, one particle in the 0th layer is considered to have come from the -1st layer for an arbitrary initial distribution). This qualitatively captures the oxidation behaviour, especially in the limits of no oxidation (where the model is exact) or full oxidation (where every atom diffusing out of the surface is lost). However, for arbitrary surface reaction barriers, we accept some error in capturing the exact value of the barrier, because we do not correctly capture particles passing through the surface multiple times. The concentration models shown in Figure 5 and Figure S14 are still qualitatively correct within the limits of the approximation, but we only give concentrations as "arbitrary units" to reflect this error. Similarly, we model the reducing effect of sputtering simply by setting the concentration at the surface layer to an arbitrary (high) value, since we have no good estimate of how much excess Ti is introduced in each sputtering step.

1. J. F. Moulder, W. F. Stickle, P. E. Sobol and K. D. Bomben, *Handbook of X-ray Photoelectron Spectroscopy*, Perkin-Elmer Corporation, 1979.
