# Supplementary figures and images for: The influence of bulk stoichiometry on near-ambient pressure reactivity of bare and Pt-loaded rutile TiO2(110)
Source: Nanoscale. 2024 Aug 29;16(38):17825–37. doi: 10.1039/d4nr01702a (PMC11381967; doi:10.1039/d4nr01702a)

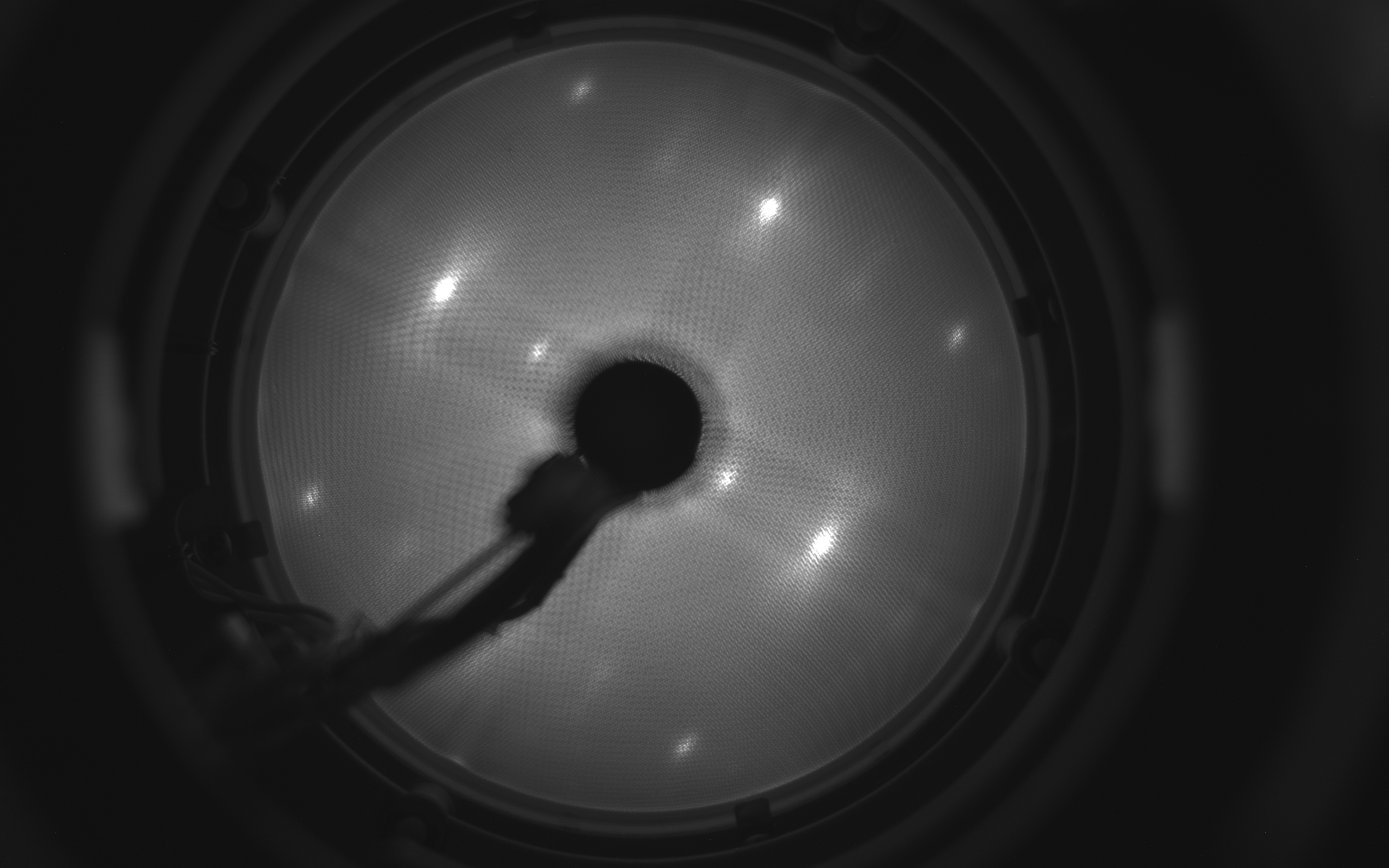

Supplement: NR-016-D4NR01702A-s002 [file NR-016-D4NR01702A-s002.zip › LEED/2023-05-31_a_6kV_70eV.png]

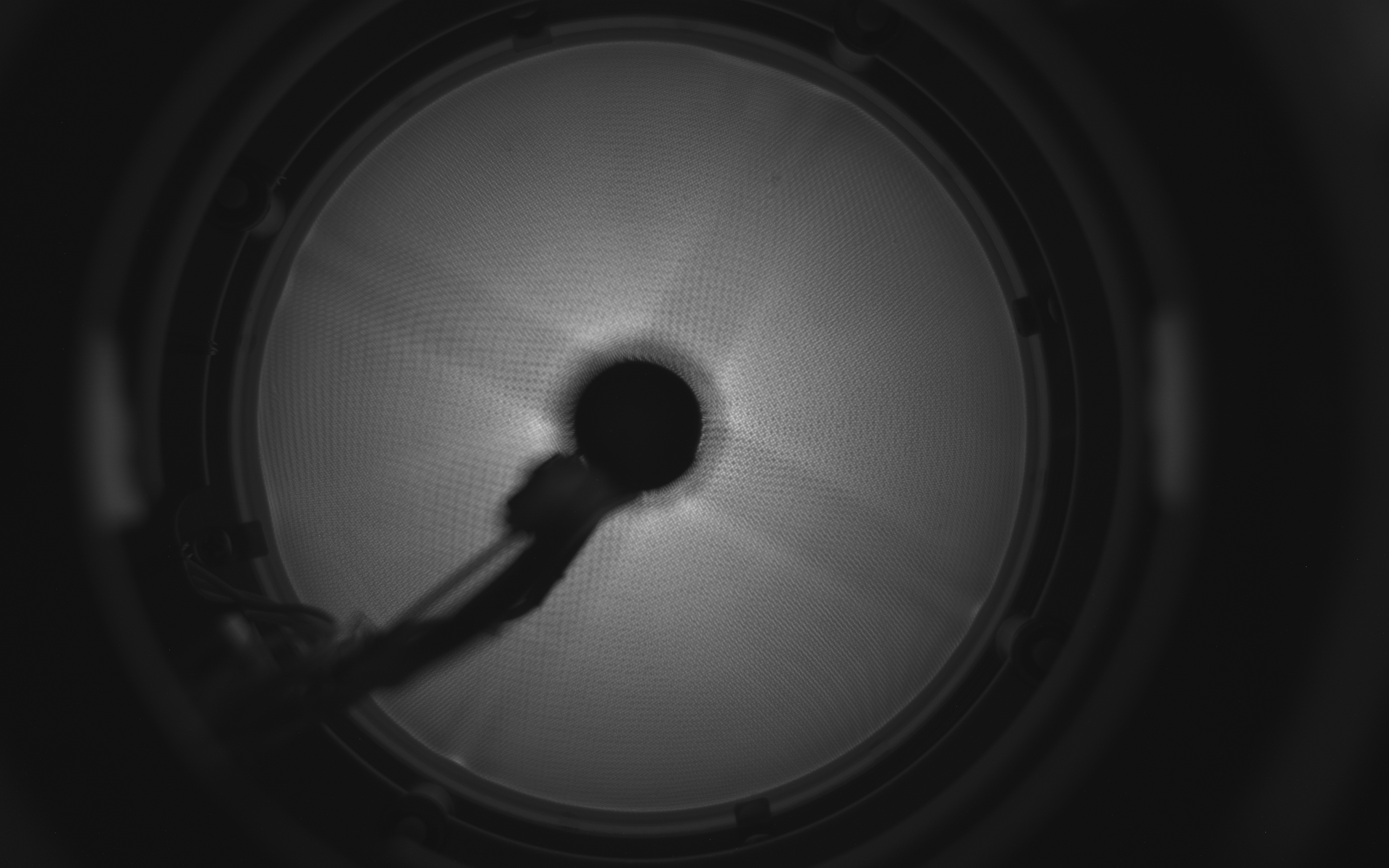

Supplement: NR-016-D4NR01702A-s002 [file NR-016-D4NR01702A-s002.zip › LEED/2023-05-31_a_6kV_70eV_ff.png]

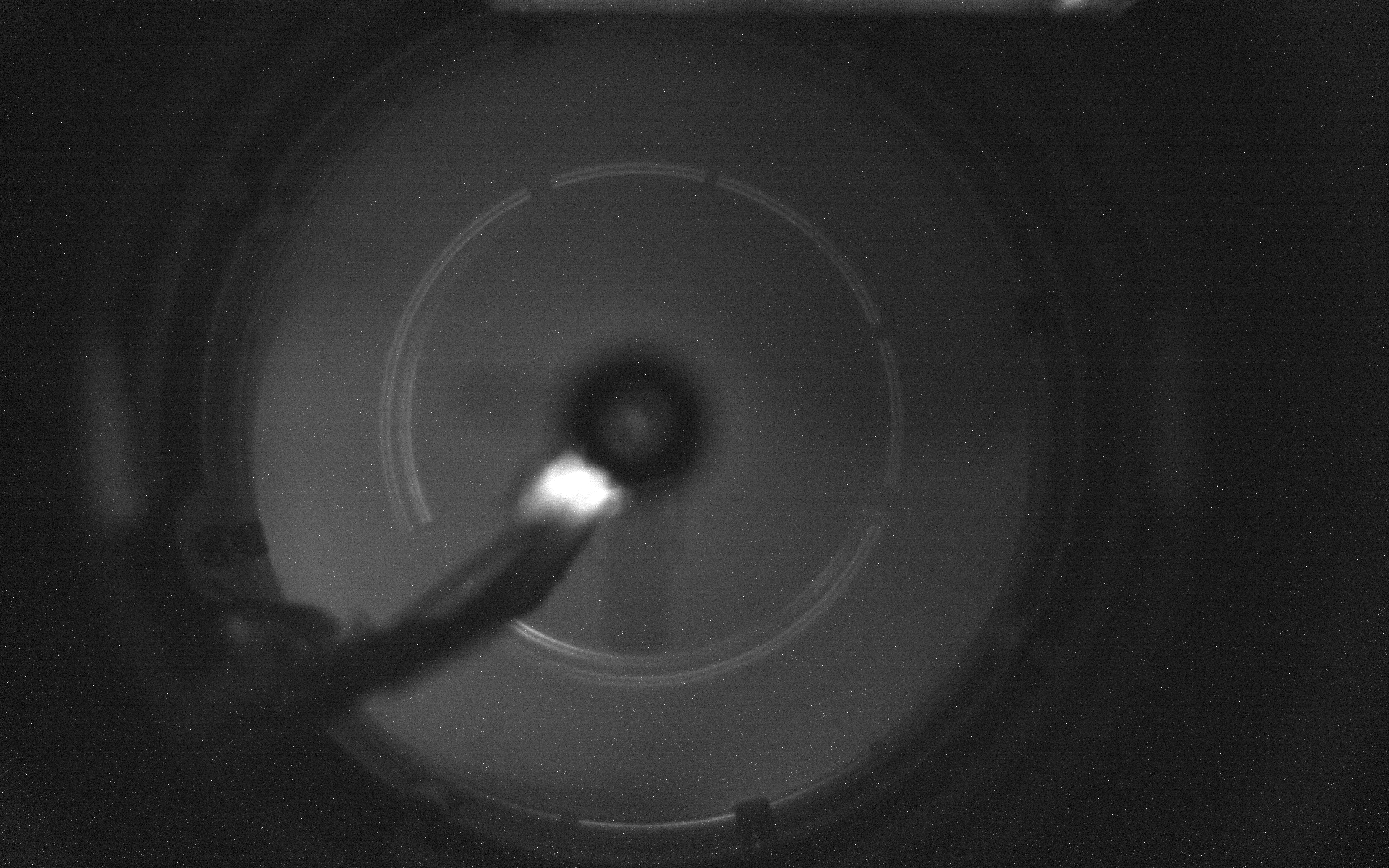

Supplement: NR-016-D4NR01702A-s002 [file NR-016-D4NR01702A-s002.zip › LEED/2023-05-31_a_df.png]

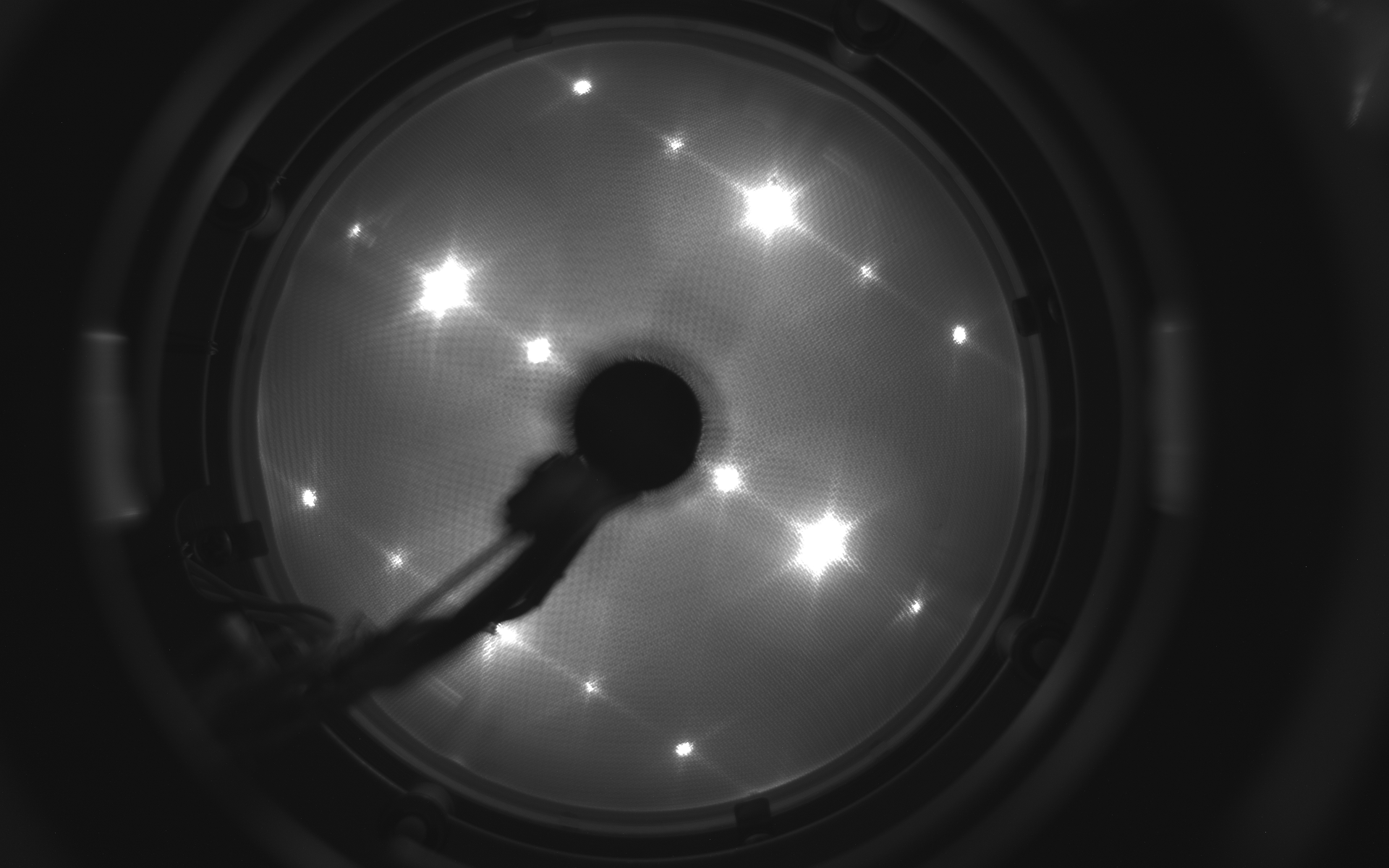

Supplement: NR-016-D4NR01702A-s002 [file NR-016-D4NR01702A-s002.zip › LEED/2023-05-31_b_6kV_70eV.png]

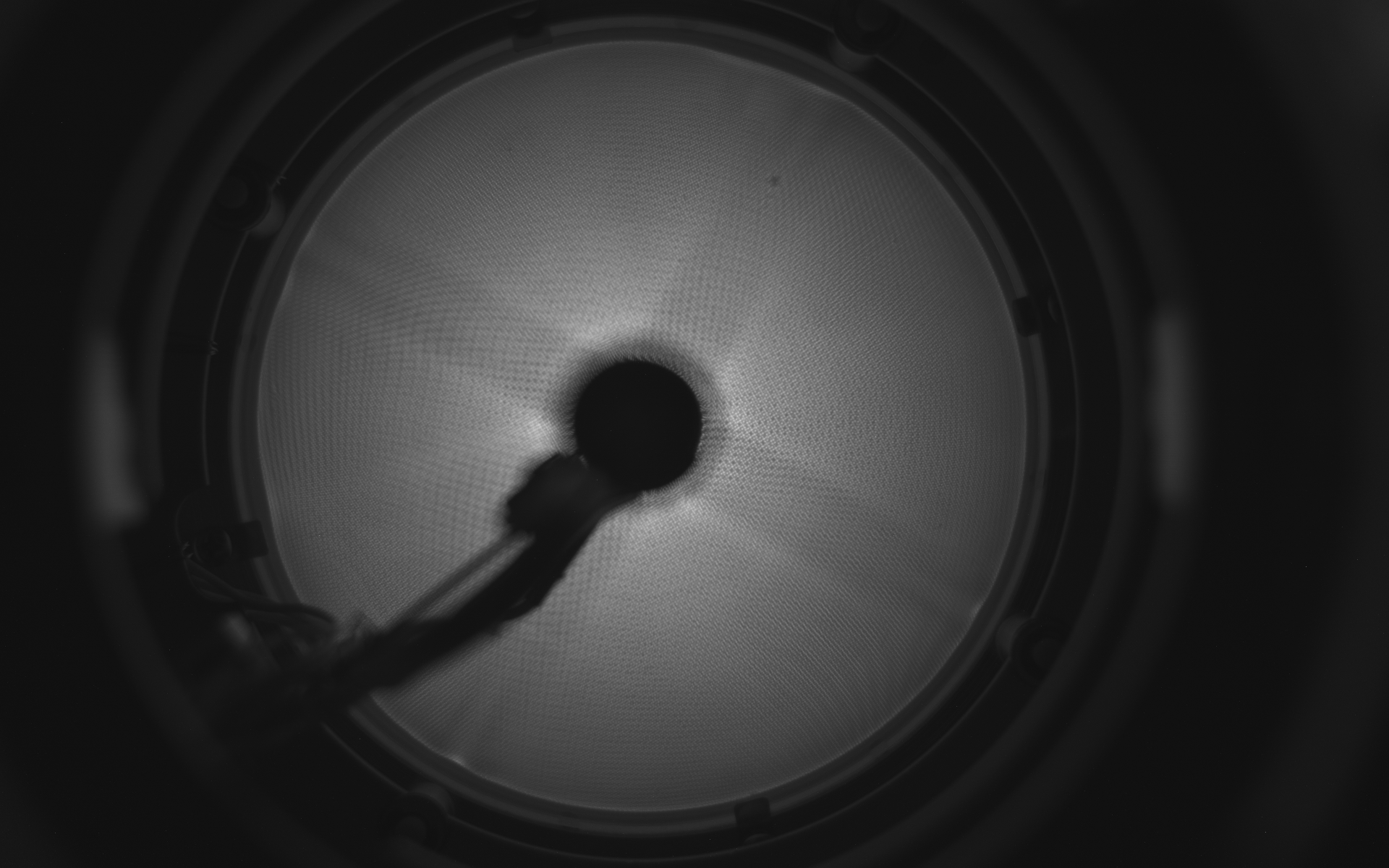

Supplement: NR-016-D4NR01702A-s002 [file NR-016-D4NR01702A-s002.zip › LEED/2023-05-31_b_6kV_70eV_ff.png]

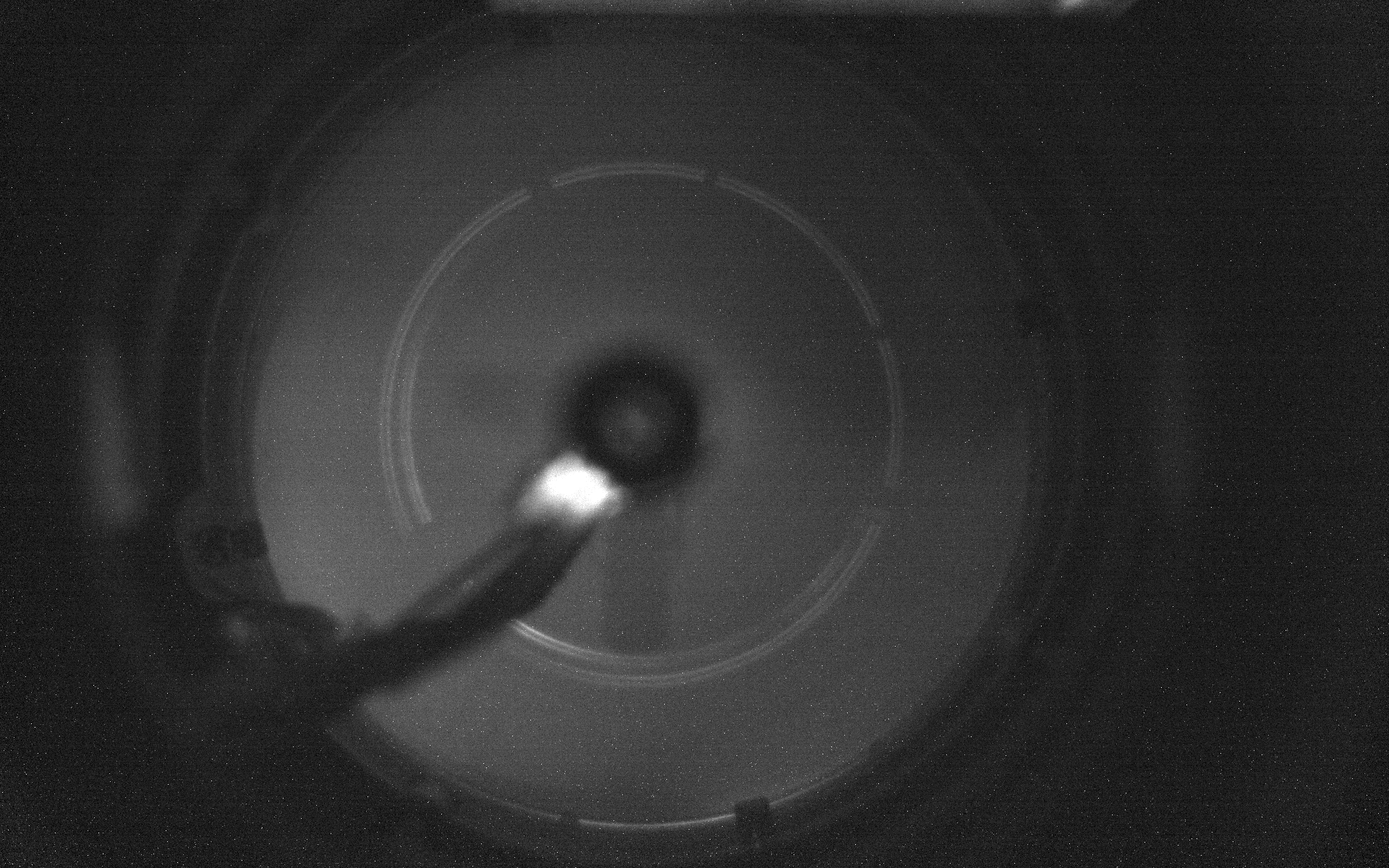

Supplement: NR-016-D4NR01702A-s002 [file NR-016-D4NR01702A-s002.zip › LEED/2023-05-31_b_df.png]
